# Contrasting impacts of competition on ecological and social trait evolution in songbirds

**DOI:** 10.1371/journal.pbio.2003563

**Published:** 2018-01-31

**Authors:** Jonathan P. Drury, Joseph A. Tobias, Kevin J. Burns, Nicholas A. Mason, Allison J. Shultz, Hélène Morlon

**Affiliations:** 1 Department of Biosciences, Durham University, Stockton Road, Durham, United Kingdom; 2 Department of Life Sciences, Imperial College London, Ascot, United Kingdom; 3 Department of Biology, San Diego State University, San Diego, California, United States of America; 4 Department of Ecology & Evolutionary Biology, Cornell University, Ithaca, New York; 5 Department of Organismic and Evolutionary Biology, Harvard University, Cambridge, Massachusetts; 6 Institut de Biologie, École Normale Supérieure, CNRS UMR 8197, Paris, France; Australian National University, Australia

## Abstract

Competition between closely related species has long been viewed as a powerful selective force that drives trait diversification, thereby generating phenotypic diversity over macroevolutionary timescales. However, although the impact of interspecific competition has been documented in a handful of iconic insular radiations, most previous studies have focused on traits involved in resource use, and few have examined the role of competition across large, continental radiations. Thus, the extent to which broad-scale patterns of phenotypic diversity are shaped by competition remain largely unclear, particularly for social traits. Here, we estimate the effect of competition between interacting lineages by applying new phylogenetic models that account for such interactions to an exceptionally complete dataset of resource-use traits and social signaling traits for the entire radiation of tanagers (Aves, Thraupidae), the largest family of songbirds. We find that interspecific competition strongly influences the evolution of traits involved in resource use, with a weaker effect on plumage signals, and very little effect on song. Our results provide compelling evidence that interspecific exploitative competition contributes to ecological trait diversification among coexisting species, even in a large continental radiation. In comparison, signal traits mediating mate choice and social competition seem to diversify under different evolutionary models, including rapid diversification in the allopatric stage of speciation.

## Author summary

Competition lies at the core of evolutionary theory and is thought to be one of the dominant processes that explain the ecological and phenotypic diversity of contemporary species. However, the extent to which competition has driven diversification at the macroevolutionary scale of large continental radiations is largely unknown. We address this question by applying a new suite of phylogenetic tools to a dataset that encompasses resource-use and social signaling traits for nearly all species of tanagers—one of the largest avian families, which contains approximately 10% of all songbirds. Using this uniquely comprehensive framework, we show that exploitative competition among lineages drives phenotypic diversification in resource-use traits (beak morphology and locomotory traits) but that the evidence that competition impacts diversification is much weaker in social signaling traits (plumage and song). Furthermore, we show that signaling traits evolve more rapidly than resource-use traits, a finding consistent with the view that traits involved in social interactions diversify during the allopatric phase of speciation, thereby reducing interspecific social competition upon secondary contact. Whereas previous research in several iconic island clades has documented the key role of competition in adaptive radiations, our research provides robust empirical evidence that interspecific competition can also structure ecological trait evolution in radiations that occur on continental scales.

## Introduction

Many explanations for how phenotypic diversity accumulates over macroevolutionary timescales assign a central role to competition between species because, in theory, such competition is expected to generate interspecific trait divergence [1–3]. Indeed, the iconic adaptive radiations of island-dwelling groups such as Galápagos finches [4,5] and Caribbean anoles [6] have highlighted the potential for interspecific competition to promote the evolution of trait differences among interacting species as envisaged by Darwin [1]. However, although there is abundant evidence that competition between species fundamentally influences a variety of ecological processes, including geographical range expansion [7,8] and community assembly [9,10], there has been little progress in quantifying the relative role of interspecific competition in trait diversification over macroevolutionary timescales outside of insular model systems. Thus, we still know surprisingly little about the overall contributions of competitive processes to trait evolution at the continental scales relevant to most of biodiversity [11].

Competition between species can impact trait evolution in several ways. For instance, the classic theory of character displacement predicts that competition between species with similar phenotypes will result in selection acting against intermediate phenotypes, resulting in interspecific trait divergence [2]. This view is most often applied to traits associated with resource use [2,12], but similar effects may extend to an array of signal traits mediating inter- and intraspecific social interactions such as mate recognition, mate attraction, and territory defense [13]. When closely related lineages interact, such traits are expected to diverge by various forms of character displacement, including reinforcement, to promote species recognition and reduce the costs of maladaptive agonistic or reproductive interactions [2,14–17]. In addition to character displacement, competition can speed up phenotypic evolution as a response to selection pressure in the presence of many competitors, leaving a pattern of positive diversity-dependence (DD) [18], or instead slow down phenotypic evolution as niches fill and ecological opportunity decreases, leaving a pattern of negative DD [19,20].

Despite the theoretical consensus that interspecific competition should influence the diversification of ecological and social traits, and the evidence for the importance of these processes at small spatial or taxonomic scales [2,21], there are several alternative processes that might influence trait evolution over large spatial and temporal scales [8]. For instance, because species typically form in geographic isolation (allopatry) and come into contact long after speciation, they may be divergent enough upon secondary contact to avoid intense competition, and thus evolution in allopatry may be more important than species interactions for explaining trait divergence [8,22]. Additionally, competition may be superseded by environmentally driven evolutionary convergence (e.g., adaptation to particular habitats) over deeper evolutionary timescales[23,24]. Moreover, the dominant form of selection on social traits may involve intraspecific processes, such as various forms of social selection [25], including sexual selection [26], thereby obscuring any effect of interspecific competition on trait evolution.

Previous studies attempting to disentangle these possibilities have met with little success because of limitations including data availability and limitations of standard, pattern-based comparative analyses [18]. A full suite of ecological and social trait data, coupled with detailed information on ecology, phylogenetic history, and geographic range data, is rarely available for diverse radiations. Moreover, it has not been methodologically possible to robustly assess the effects of interspecific competition against alternative hypotheses for the evolution of different classes of traits [11,18]. Predicting that competition will drive coexisting species to be more phenotypically divergent than non-coexisting ones, investigators have tested for a correlation between sympatry and trait dissimilarity [27]. However, such approaches often fail to detect the impact of competition on trait evolution when it exists, precluding robust conclusions [18].

## Results and discussion

**Fig 1:**
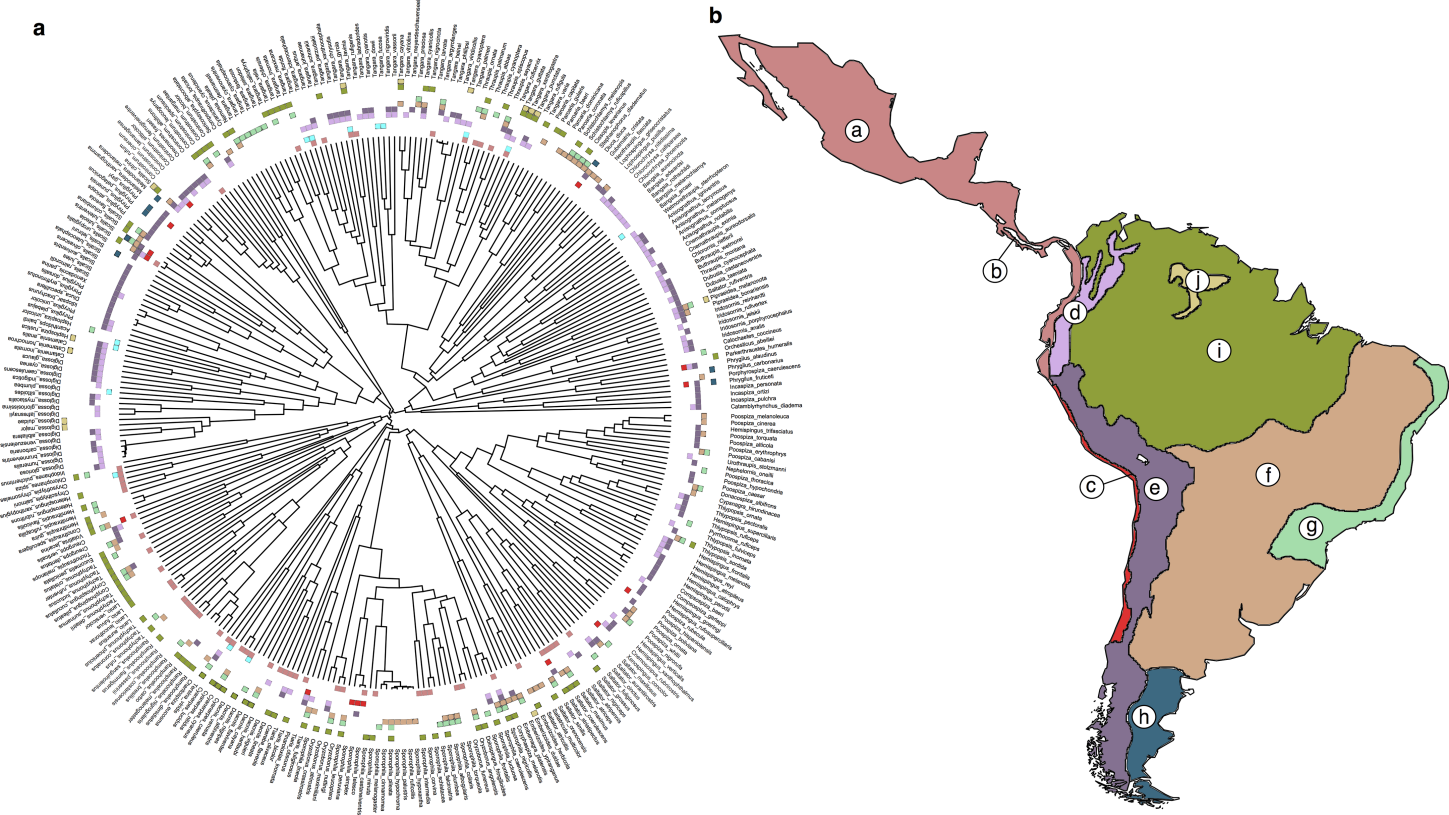
Biogeography of tanagers (Thraupidae). (A), Phylogeny of study species, with colors at tips denoting geographical distributions corresponding to the areas shown in (B). (B), Map of the regions used for ancestral range estimation. Key to regions as defined by ref. [37]: a, Gulf-Caribbean slope, Pacific arid slope, and Chocó lowlands; b, Chiriquí-Darién highlands; c, subtropical Pacific; d, northern Andes; e, central and southern Andes; f, central South America and Pampas; g, Atlantic forest; h, Patagonia; i, Amazonia (north and south) and northern South America; j, Tepuis.

In this study, we fit recently developed phylogenetic models designed for assessing the impact of interspecific competition on trait evolution [28] to comprehensive estimates of resource-use [29,30], plumage [31], and song traits [32] for nearly all (*n* = 323) continental lineages of tanagers, the largest family of songbirds (Fig 1A and 1B). In combination, these datasets provide uniquely detailed quantitative information on resource-use and signal traits for nearly 10% of all songbirds. We compared the statistical support for three phylogenetic models incorporating interspecific competition—matching competition (MC), linear diversity-dependent model (DD_lin_), and exponential diversity-dependent model (DD_exp_) [28], accounting for estimated ancestral range overlap [33]—to two models that are neutral to the influence of interspecific competition: Brownian motion (BM) and Ornstein-Uhlenbeck (OU) models [34,35]. By fitting these process-based models, we can explicitly test hypotheses about the relative importance of competition within the same nested framework and across different subgroups of potentially interacting lineages grouped according to diet [29], habitat [36], and year-round territoriality [36] without having to rely on pattern-based approaches that can often lead to misleading inferences about competition [18].

**Fig 2:**
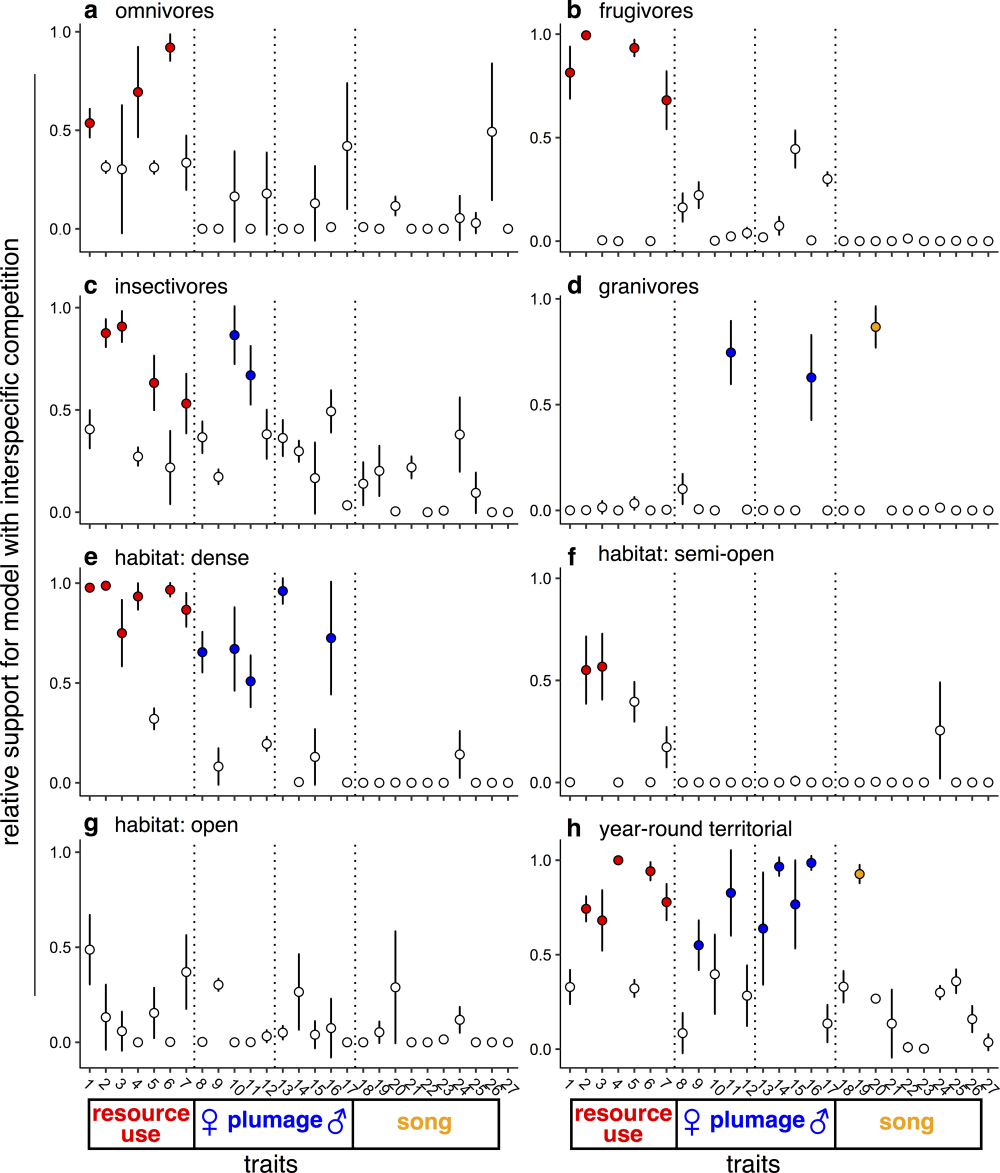
Plotted is the relative support for the best model that incorporates interspecific competition (MC, DD_lin_, or DD_exp_) when compared to the best model that does not incorporate interspecific competition (BM or OU) ± the standard deviation across fits to the sample of stochastic maps for each trait (see S4 Data). Filled points represent model fit cases in which the mean relative support for a model with competition is higher than for a model excluding competition (i.e., relative support > 0.5). Species were grouped by diet (A-D), habitat (E-G), and year-round territoriality (H). Trait numbers (1–27) are defined in S1 Table. BM, Brownian motion; DD_exp_, exponential diversity-dependent model; DD_lin_, linear diversity-dependent model; MC, matching competition; OU, Ornstein-Uhlenbeck.

The results reveal that many resource-use traits in tanagers are shaped by interspecific competition—diversity-dependent or MC models were the best fitting models for one or more of these traits in nearly every subgroup analyzed (Fig 2, S6 Fig, S7 Fig, S3 Table). The only exceptions were granivorous and open-habitat species, both of which tend to be either weakly territorial or nonterritorial because they are adapted to tracking patchy or ephemeral food sources [36,38]. This evidence is consistent with classical models that emphasize interspecific competition as one of the dominant evolutionary forces in nature [12]. Contrary to Simpson’s adaptive zone model [39], in which phenotypic diversification slows down as species accumulate and fill ecological space, we find more traits under positive than negative DD, suggesting that competition fosters diversification (S6 Fig, S3 Table).

For social traits, we again found evidence that plumage trait evolution was shaped by interspecific competition, though to a lesser extent than for resource-use traits (Fig 2, S6 Fig, S7 Fig, S3 Table). Again, positive DD found frequent support (S6 Fig, S3 Table). Of all subgroups of potential competitors analyzed, plumage traits were most consistently influenced by competition in species that defend year-round territories, suggesting that although there is no straightforward cladewide effect of interspecific competition, interactions between lineages may affect plumage evolution at finer scales. Perhaps unsurprisingly—given that females of many tanager species are similarly ornamented to males or even indistinguishable to human observers [40]—competition influenced both male and female plumage traits roughly equally (Fig 2). Indeed, in some instances, female plumage traits were more strongly affected than those of males (e.g., Fig 2C, S7 Fig, S3 Table), consistent with observations that female ornamentation is closely tied to social competition for resources [36]. In contrast, song evolution did not appear to be influenced by interspecific competition at any scale, suggesting that other processes predominate in shaping the evolutionary trajectories of song traits (S3 Table).

**Fig 3:**
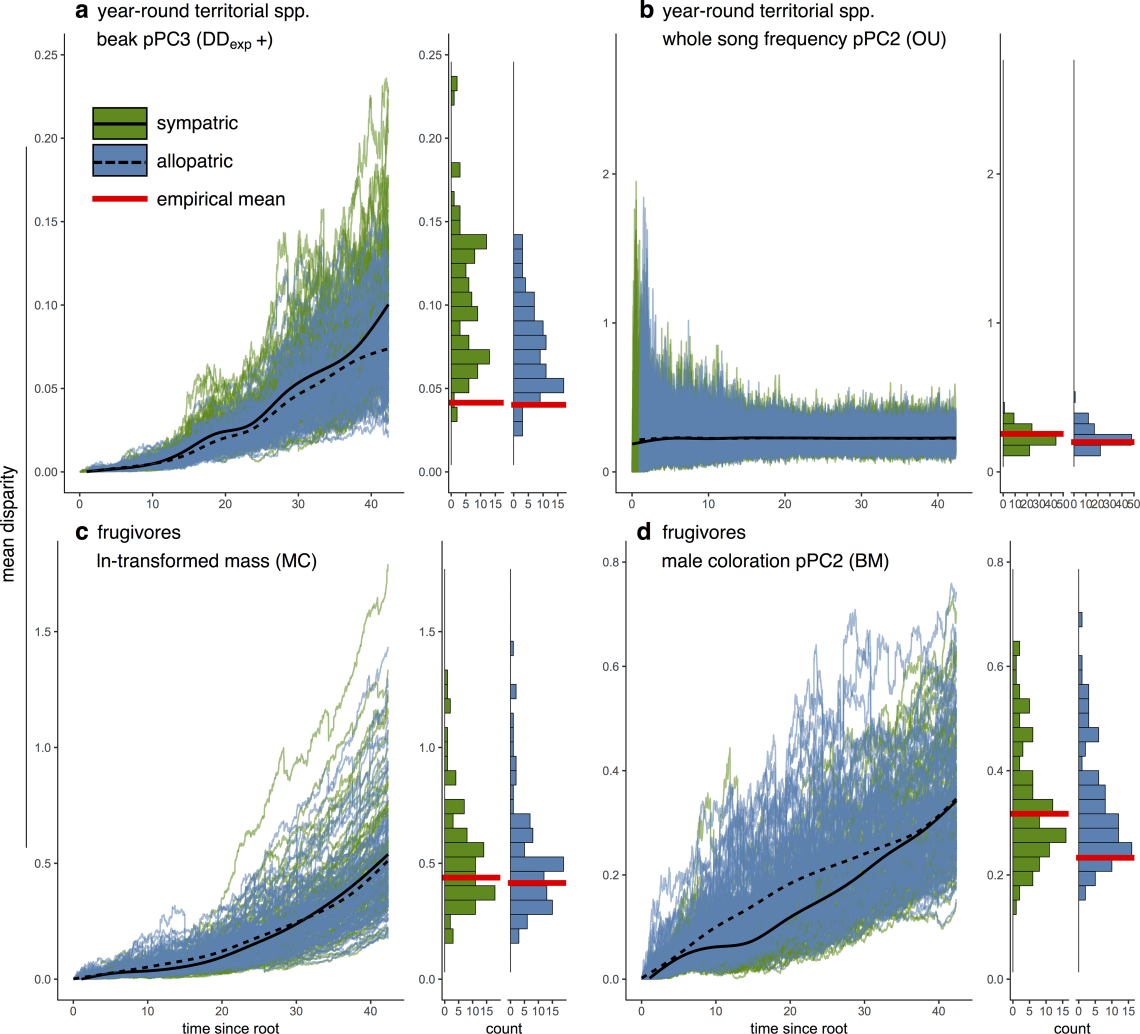
Trait disparity through time between allopatric and sympatric lineages for the following four representative scenarios: (A) Beak pPC3 for year-round territorial taxa (trait number 4, S1 Table), (B) whole song frequency pPC2 for year-round territorial taxa (trait number 22, S1 Table), (C) ln(mass) for frugivores (trait number 1), and (D) male coloration pPC2 for frugivores (trait number 17, S1 Table). The best-supported model is indicated. Each panel depicts the mean disparity, calculated as the average squared Euclidean distance, between allopatric lineages and between sympatric lineages through time in 100 datasets simulated with the MLE parameter values for the best-fit model (left) and the distribution of mean sympatric and allopatric disparity values at the tips (right). Red lines depict the mean disparity values in the empirical datasets (see S5 Data). BM, Brownian motion; DD_exp_, exponential diversity-dependent model; ln(mass), log-transformed mass; MC, matching competition; MLE, maximum likelihood estimate; OU, Ornstein-Uhlenbeck; pPC2, phylogenetic principal component 2; pPC3, phylogenetic principal component 3.

In order to illustrate patterns resulting from differences in the processes of evolution inferred from our model fitting procedure, we reported the best-fit models’ predictions for the accumulation of disparity through time between allopatric and sympatric lineages for two resource-use traits and two signaling traits supporting different models (Fig 3). There are visible differences in the way that disparity accumulates through time depending on the evolutionary process. For example, disparity accumulates in pulses when DD_exp_ is the best-fit model, with pulses corresponding to jumps in the number of interacting clades (Fig 3A). In contrast, under the MC model, disparity accumulates gradually until near the present (Fig 3C). These differences lead to distinct between-species covariances that process-based models can detect. In contrast, none of these processes (whether they include competition or not) produce marked differences in the disparity of sympatric versus allopatric lineages. There can be some slight differences in the mean disparity between sympatric and allopatric lineages (e.g. Fig 3A), but the variation across simulations is such that we would in general not expect to detect significant differences. This is likely because in large clades, the networks of allopatry and sympatry are complex and involve multiple interacting species, such that divergence between sympatric taxa is not independent of divergence between allopatric taxa as in simple two-species models. Thus, process-based models detect signals of competition in the data that are not apparent in simple comparisons of present-day patterns of divergence between sympatric and allopatric species, which are often used as tests of character displacement [18].

A potential reason for weaker effects in social signals is that trait divergence is not the only possible outcome of competition. When species compete strongly for access to the same resources, selection may actually favor convergence in signal traits that mediate territorial interactions [17]. Thus, the broad-scale signature of interspecific competition may be sympatric convergence in songs and plumage [11], a pattern which none of the process-based models of competition used here were designed to detect. To address the possibility that competition leads to signal trait convergence in tanagers, we used phylogenetic simulations to test for sympatric convergence while controlling for similarity in diet and habitat, which previous research demonstrates is the best method for detecting convergent character displacement [18]. These analyses did not identify patterns of sympatric convergence in either plumage or song traits, except for weak convergent trends in some restricted scenarios for insectivores and species that live in semi-open habitats (S4 Table). We can thus rule out the possibility of clade-wide sympatric convergence in signal traits of tanagers, but acknowledge that both divergence and convergence may occur in such traits across different species pairs, obscuring patterns emerging from heterogeneous effects of interspecific competition in clade-wide analyses.

**Fig 4:**
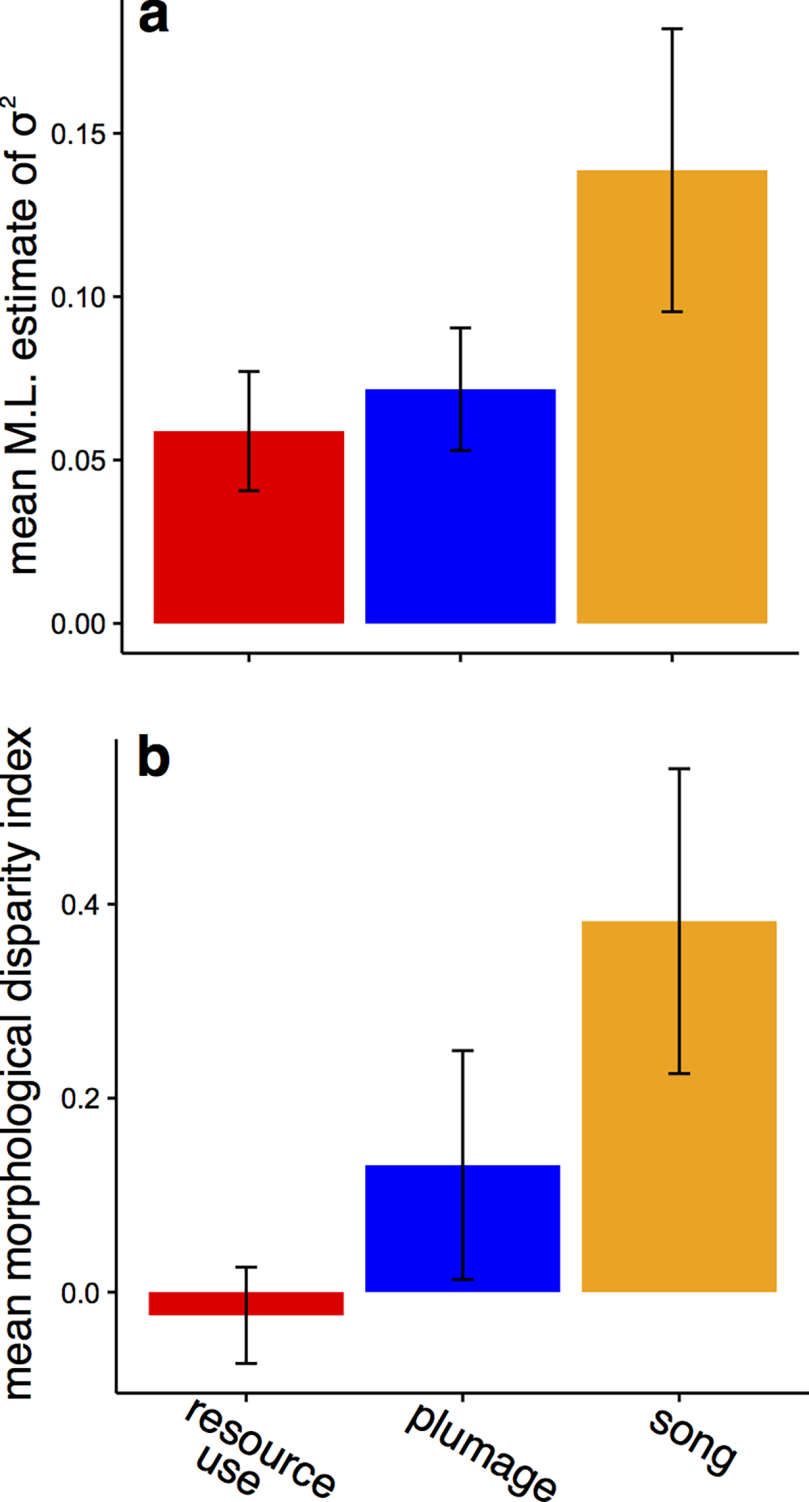
The tempo of ecological and social trait evolution. (A) Mean MLE of the σ^2^ (evolutionary rate) parameter from the BM model fit to standardized trait values show that song traits evolve more rapidly than resource-use or plumage traits in either sex. Plotted are mean ± SD of mean values for each class of trait from fits across 100 posterior trees. (B) Mean MDI estimates similarly indicate that song traits accumulate within-clade disparity more rapidly than resource-use or plumage traits in either sex. Plotted are mean ± SD of mean values for each class of trait calculated across 100 posterior trees (see S6 Data). BM, Brownian motion; MDI, morphological disparity index; ML, maximum likelihood.

Another possible explanation for our findings that interspecific competition does not affect plumage and song traits as intensely as resource-use traits is that signal evolution is more rapid than the evolution of resource-use traits, thus precluding wasteful interspecific interactions upon secondary contact [25,26,41]. We found support for this hypothesis in the accelerated divergence of song traits, as they evolved more quickly than resource-use traits (Fig 4A, S9 Fig, S10 Fig, S11 Fig, S12 Fig, S13 Fig, S14 Fig and S15 Fig; ANOVA test for effect of trait type [resource use, plumage, versus song] on BM σ^2^ estimates for *z*-transformed trait values, mean across 100 posterior trees: F_2,24_ = 19.79, *p* < 0.001; ANOVA test for effect of trait type on log-transformed felsen values, mean across 100 posterior trees: F_2,24_ = 9.72, *p* < 0.001; ANOVA test for effect of trait type on ln-transformed BM σ^2^ estimates for untransformed trait values incorporating measurement error (ME), mean across 100 posterior trees: F_2,24_ = 8.07, *p* = 0.002) and accumulated disparity faster than other traits (Fig 4B, S9 Fig, S13 Fig and S16 Fig; ANOVA test for effect of trait type on morphological disparity index (MDI), mean across 100 posterior trees: F_3,23_ = 13.99, *p* < 0.001). These results are consistent with previous studies in tanagers showing that rapid song evolution [42] in the initial stages of speciation—likely made possible because tanager songs are shaped by learning and thus subject to cultural evolution—results in complete species isolation following secondary contact [43], generating coincident bursts of song evolution and speciation [44]. In other words, song-mediated species recognition may not arise through interspecific competition, but earlier, during a period of geographical isolation in allopatric ranges [25]. In contrast with song traits, plumage traits exhibit intermediate rates of trait evolution and accumulation of disparity, though these patterns do not differ markedly from resource-use traits (Fig 4B, S9 Fig, S13 Fig and S16 Fig). We note that the differences between song and plumage trait evolution may arise because greater constraints on plumage evolution prevent unbounded divergence (e.g., selection for plumage crypsis opposing the effects of sexual selection [31], S4 Table). Although we did not find a direct negative relationship between evolutionary rate per se and evidence for competition (S17 Fig), there is a trend in the predicted direction, and together with estimates of disparity and divergence, our results are consistent with a model of evolution in which the impact of interspecific competition is negatively related to evolutionary rates: traits either evolve rapidly (e.g., under social selection) and escape the effect of competition upon secondary contact, or they evolve slowly and thus are subject to the effect of interspecific competition in sympatry.

We note that there are other possible reasons for why we did not detect a consistent effect of competition on traits involved in social interactions. For one, competitively mediated diversification may be better described as proceeding in a multivariate fashion or along trait axes not measured here. Also, diversification of traits involved in social interactions are also impacted by the recognition of those signals by receivers [45–47], which is particularly challenging for large-scale comparative analyses. Finally, the fact that traits involved in social interactions tend to be more intraspecifically variable may make it more likely for OU models to be erroneously selected even if competition played an important role in the evolutionary history of a trait [48].

More generally, intraspecific variability and ME can bias inferences from phylogenetic comparative methods [39]. In particular, ME increases variance at the tips and can lead to a spurious support for models in which evolution is faster in the recent past, such as OU or positive DD models. Therefore, MEs could either spuriously decrease or increase support for competition models (decrease if giving support for OU, increase if giving support to positive DD). We did find a negative relationship between ME and support for competition models (S5 Table, S18 Fig). This relationship was driven by the low support for competition models in song traits that happened to display high intraspecific variability (S18 Fig); after controlling for this relationship we still found that resource-use traits contain a stronger signature of interspecific competition (S5 Table). In addition, the effect of ME disappeared if we considered only datasets in which ME was reasonably low (S5 Table). There was an effect of ME on the parameter estimate of the DD_exp_ model, with more positive values being more likely with increasing ME (S6 Table). We note however that we found high support for positive DD even in analyses with low ME (S8 Fig), suggesting that there are some cases where positive DD occurs. In aggregate, therefore, although ME could be responsible for some of the parameter estimates of—but not the statistical support for—positive DD, it is unlikely to drive our main result that interspecific interactions are less important for structuring the diversification of social traits than resource-use traits.

With available methods, we cannot rule out the possibility that interspecific competition acts on species assembly by favoring the coexistence of phenotypically distinct lineages [7]. The patterns we describe may therefore arise through a process of ecological sorting in addition to, or instead of, evolutionary divergence. Regardless, our findings clearly establish that the effects of competition are not limited to insular model systems [4,6] but instead can drive the distribution of resource-use traits in large, continental radiations. Traits involved in social signaling—especially song—diverge by different or additional processes, with interspecific competition perhaps subordinate to intraspecific processes. Overall, these findings reinforce the view that competition structures evolutionary radiations, even at large temporal and spatial scales, and highlight the varying effects of competition on different traits.

## Materials and methods

### Phylogeny and trait data

We derived a maximum clade credibility phylogenetic tree for the entire tanager clade (*n* = 355 spp.) [49] from the posterior distribution of trees constructed in BEAST [50] using two mitochondrial markers (Cyt-b, ND2) and four nuclear markers (RAG1, FGB-I5, MB-I2, and AC01-I10). We also used the posterior distribution of trees to assess the robustness of our results to topological and dating uncertainty (S8 Fig). To simplify biogeographic reconstructions (see more details in the Subsetting likely interactions section below), and to focus on continental-scale processes, we excluded insular taxa and taxa lacking biogeographic data (*n* = 32), leaving a dataset restricted to continental taxa (*n* = 323).

We compiled measurements of ecological and social traits for as many species as possible. For resource-use traits, we collected data on beak length, width, and depth, as well as wing, tarsus, and tail length from museum specimens of tanagers (*n* = 319 species) using established procedures [30,51]. We also included published data on body mass (*n* = 322) [29]. For social traits, we compiled data on both visual signals (plumage coloration) and acoustic signals (song). Variation in plumage coloration were extracted from reflectance spectra measured separately on males (*n* = 312) and females (*n* = 314), with species differences calculated by comparing between overall coloration patterns as well as between particular plumage patches [31,52]. Acoustic data included song tempo (*n* = 294), song frequency (*n* = 294), and the temporal changes in frequency throughout songs (*n* = 287) [32,53]. All traits were measured on several individuals of each species where possible (mean ± standard deviation: resource-use traits = 5.35 ± 7.69 individuals, male plumage coloration = 4.27 ± 1.17, female plumage = 3.84 ± 1.32, song = 8.53 ± 7.24).

We constructed phylogenetic principal component axes in phytools [54,55]. Apart from two song variables (frequency and amplitude slopes), all data were ln-transformed prior to construction of the axes, and trait data for all taxa, including island taxa and taxa without biogeographic data, were included in the construction of the pPC axes. Rather than compiling all data into a single set of pPC axes, we grouped variables that describe functional variation in birds. In total, we constructed nine sets of pPC axes describing variation in resource-use (axes for beak morphology and locomotory traits), plumage (axes for male and female plumage coloration and complexity), and song (song tempo, song frequency, and note frequency) (S1 Table, S2 Table). We used pPCs, rather than untransformed raw trait data, as previous work has shown that these character axes describe functionally relevant traits [30]. Although we acknowledge the circularity of using pPCs in comparative analysis of traits, we note that using phylogenetically informed principal components and including all of the resulting axes should minimize the impact of known statistical issues arising from using PCs in comparative analyses [56]. In addition to the pPC axes, we also ran analyses on ln-transformed mass, which is a commonly used proxy for resource use in comparative studies [57].

### Subsetting likely interactions

Because only species that coexist can interact, models that incorporate species interactions require identifying subsets of sympatric species [28]. To assign each lineage to a biogeographic region, we began by determining the region for each lineage as defined by ref. [37]. To ensure that the ancestral range estimation models were not too complex and thus too large to fit, we then collapsed these regions with >65% overlapping species and removed island taxa (Galápagos and other equatorial Pacific islands, south Atlantic islands, and Caribbean islands; *n* = 30), as well as two species endemic to the Madrean highlands, resulting in 10 final biogeographic regions (see Fig 1). In total, there are 323 species for which we had both phylogenetic and regional data. In BioGeoBears [33], we fit the dispersal-extinction-cladogenesis model of range evolution to the observed ranges of species. In this model, ranges change anagenetically as a function of dispersal and local extinction parameters and cladogenetically as a function of several range inheritance scenarios (see additional details in refs. [33,58]). Any attempt at ancestral range estimation over large macroevolutionary and spatial scales is bound to return a crude estimate of lineage presence and absence through time. To incorporate uncertainty in these reconstructions, we used the maximum likelihood parameter values for the DEC model to generate a bank of stochastic maps [59] using the function runBSM, each representing a hypothesis about where each lineage existed at a given point in time. These stochastic maps are largely congruent in which lineages they assign as sympatric (>75% congruent in the most recent 35 My) and thus largely represent uncertainty at much deeper timescales (S1 Fig). We also note that there is no reason why error in ancestral range estimation would impact distinct traits differently.

Additionally, competition is only likely between species that use similar resources or that come into close contact within their range. To estimate the likelihood of competition, we delineated three sets of potential competitors. We defined one set of species based on their basic habitat preferences [36] (S2 Fig). Specifically, each lineage was classified as occurring in dense (*n* = 128), semi-open (*n* = 143), or open habitats (*n* = 49). We defined another set based on diet using the EltonTraits database [29]. Each species was assigned to one of four of the following diet categories: invertebrate specialist (≥60% invertebrate diet, *n* = 80), frugivore (≥60% fruit diet, *n* = 59), granivore (≥60% seed diet, *n* = 66), or omnivore (<60% of either category, *n* = 115) (S3 Fig). We removed two species of nectarivores (*Coereba flaveola* and *Diglossa gloriosissima*) prior to running analyses. Following Tobias et al.[36], we defined a third set of species that defend permanent (year-round) territories (*n* = 35) (S4 Fig), on the grounds that these lineages are more aggressive, and thus more likely to interact in agonistic contexts than lineages defending only breeding territories. For each category, we fit *Mk* models of discrete character evolution to the tree, allowing transitions between each subcategory to occur at different rates. We then used the maximum likelihood Q matrix for each category to construct a bank of 50 stochastic maps using make.simmap in phytools [55], in which each stochastic map is a hypothesis about which guild each lineage belonged to at any given moment in evolutionary history.

Stochastic maps of biogeography were combined with stochastic maps of set membership to create a series of interaction matrices (**A**) defining which lineages are able to interact at each point along the phylogeny (S5 Fig). Thus, for a given time (*t*), if lineages *i* and *j* are both estimated to occur in the same region and to belong to the same set (e.g., insectivores), **A**(*t*)_*i*,*j*_ = 1. Otherwise, **A**(*t*)_*i*,*j*_ = 0. We then fit datasets to these trimmed trees by specifying that only lineages that are both sympatric and belong to the focal subgrouping (e.g., insectivores) can interact (lineages of nonfocal subgroupings evolve as if in allopatry, i.e., via BM). After calculating the expected variance-covariance matrix under this model, we then trimmed any remaining tips that do not belong to the focal subgrouping for subsequent likelihood calculation.

Although different partitions varied in the number of taxa with trait data (range = 32–143 spp.), there was no effect of sample size on the mean relative support for a model including interspecific competition (as defined in Fig 2; linear regression: effect of sample size on relative support index: *t* = −0.65, *p* = 0.52).

### Model fitting

Rather than isolate a subset of pPC axes on which to run analyses, which can bias trait evolution models [60], we ran our analyses on all 26 pPC axes and body mass data. We ran the following two models that did not account for interspecific competition: the BM drift model [34] and the OU model of adaptation to an optimum trait value [35]. We also ran the following three models that accounted for interspecific competition: the MC model, in which the trait values of competing lineages are repelled from one another [28,61], and two diversity dependent models (DD_lin_ and DD_exp_), in which evolutionary rates vary as a positive or negative linear or exponential function of the number of lineages in the reconstructed phylogeny [20,28]. In clades with high levels of extinction, inferences made on contemporary species can be misleading. Diversity-dependent models, in particular, can generate biased inferences when the number of reconstructed lineages does not accurately reflect the true historical diversity (e.g., if there are many extinct lineages the magnitude of slope parameters are underestimated [28]). Thus, we first verified that model estimates of diversity through time did not deviate from the observed number of reconstructed lineages through time (see *Diversification analyses*, below). We fitted BM and OU models using fitContinuous in geiger [62], and MC and DD models with interaction matrices (described above) using fit_t_comp in RPANDA [63]. We constrained the *S* parameter of the MC model to be negative to stabilize likelihood optimization, but we allowed the rate parameters of the DD models to be either positive or negative (reflecting rate increases [positive DD] or rate decreases [negative DD] with increasing species richness, respectively). For each species category, we selected the best model as the model with lowest corrected Akaike Information Criterion (AICc). The model selection procedure used here has reasonable power and Type I error rates [28], and simulations under a model of character displacement show that both MC and DD_exp_ can detect signatures of character displacement [18]. We also computed the Akaike weight of each model (denoted wi) and measured the relative support for a model with interspecific competition (MC, DD_lin_, or DD_exp_) as the relative Akaike weight of the best model with competition when compared to the best model without competition: max(MC_wi_, DD_lin_wi_, DD_exp_wi_)/((max(BM_wi_,OU_wi_)+max(MC_wi_, DD_lin_wi_, DD_exp_wi_)).

A series of other trait evolution models have been developed in the literature (reviewed in [64]), including models with evolutionary rates that vary continuously with time [57] or variation in environmental variables [65], with discrete shifts [66], and more complex OU models in which large clades are given their own rates and optima [24,67]. We did not consider the former because we wanted to focus on process-based models rather than phenomenological models [28], nor the latter because they serve to test other types of hypotheses, such as large-scale convergent evolution [23,24].

### Diversification analyses

To assess whether model estimates of diversity through time and the number of lineages present in the reconstructed phylogeny are concordant, we fit several diversification models to the MCC tree using fit_bd in RPANDA [63]. Specifically, we fit (1) a pure-birth model with constant speciation and no extinction, (2) a pure-birth model with exponential variation in speciation and no extinction, and (3) a birth-death model with exponential variation in speciation and constant extinction. Both models with exponential variation in speciation outperformed the pure-birth constant rate model (model 2 versus 1, likelihood ratio = 40.97, *p* < 0.001; model 3 versus 1, likelihood ratio = 44.17, *p* < 0.001), but the model with extinction did not outperform the simpler model without extinction (model 3 versus 2, likelihood ratio = 3.20, *p* = 0.07). Thus, because extinction is estimated to be negligible, we conclude that standard diversity-dependent models that rely on the number of lineages in the reconstructed phylogeny are adequate for our analyses. We acknowledge that the lack of significant evidence for extinction likely represents the well-known low power to detect extinction in reconstructed phylogenies rather than a true absence of extinction [68] (see also discussion in [69]). However, there is no reason to expect that this would systematically bias inferences for one trait type (e.g., resource-use traits) or guild (e.g., frugivores) and not others.

### Regression-based analyses

Although interspecific competition should drive diversification of resource-use traits, exploitative competition between species can actually result in adaptive convergence in signal traits; if the benefits to an individual of excluding heterospecifics from their territory are similar to the benefits of excluding conspecifics, then selection may favor convergence in traits that are used to mediate territorial interactions [17,70]. However, the process-based models of interspecific competition are not designed to detect trait convergence resulting from interspecific interactions [18]. Therefore, to test whether interspecific competition leads to trait convergence in competing lineages, we coupled linear regressions with phylogenetic simulations to test for a pattern of sympatric convergence [18,71].

In these models, we tested for convergence in (1) female plumage coloration pPC axes, (2) male plumage coloration pPC axes, and (3) song pPC axes. For each of these three groups of traits, we created a dissimilarity index by calculating the square-root transformed Euclidean phenotypic distance between all relevant pPC axes. To quantify sympatry, we calculated between-species range overlap using range data from BirdLife International and NatureServe [72]. We calculated the Szymkiewicz-Simpson coefficient (the area of overlap divided by the area occupied by the species with the smaller range) and created four indices of sympatry (one each for Szymkiewicz-Simpson coefficient threshold values of 5%, 20%, 50%, and 80%) [73]. Because convergence in sympatry can also result from selection favoring particular phenotypes in similar environments, we also included predictor variables indicating whether species pairs were found in the same habitat type or had the same diet. If, after controlling for similarity in habitat or diet, sympatry was negatively correlated with the dissimilarity index, this would be consistent with character convergence resulting from interspecific interactions.

To correct for phylogenetic nonindependence in these pairwise datasets, we conducted a phylogenetic simulation, which is more reliable than other methods for controlling for phylogenetic structure [18]. We simulated 5,000 datasets for each of the 27 traits using the ML parameter estimates for both (1) the BM model and (2) the OU model. We then conducted the same regression models on Euclidean distances between each of these simulated datasets to produce a phylogenetically informed null distribution of test statistics against which to compare our nonphylogenetic test statistic. We then calculated a two-tailed *p*-value using this distribution, which we subsequently corrected for multiple testing (using a critical value of 0.0125 because we conducted analyses on four sympatry indices [i.e., 0.05/4]).

### Tempo of trait evolution

To estimate rates of evolution, we fitted BM models to the MCC tree and 100 posterior trees and the most complete trait datasets available (i.e., incorporating island and continental taxa, *n* = 313–353, see S2 Table). Because BM rate parameters are sensitive to changes in the scale of the trait values, we transformed all variables into *z*-scores so that all traits were on the same scale. We then ran ANOVAs to test whether the trait type (i.e., resource-use, female plumage, male plumage, and song) explained variation in the maximum likelihood estimate (MLE) of the BM rate parameter (σ^2^) on each of the 100 trees.

In addition to calculating the rates of trait evolution as the MLE of σ^2^ in BM models, we calculated two other indices of evolutionary rates: (1) “felsens,” a measurement of divergence between sister taxa scaled by their divergence time [74], and (2) BM fits to untransformed data, incorporating ME as the standard error of each individual trait measurement where available (unknown values were then estimated directly as an additional parameter), in geiger [62]. We then fit ANOVAs to the ln-transformed felsen value and the ln-transformed estimate of the BM rate parameter (σ^2^), as above.

We further examined the tempo of trait evolution by analyzing how disparity accumulates through time for each trait [75]. Using the dtt function in geiger [62] with the average squared Euclidean distance among *z*-transformed trait values, we simulated 1,000 BM datasets to create a null distribution for the MCC tree and each of 100 posterior trees. We then calculated the MDI as the area between the null and observed disparity-through-time curves for each phylogeny, such that large MDI values indicate rapid and sustained accumulation of within-clade disparity greater than that expected under BM. As with rates of trait evolution, we ran ANOVAs on each posterior tree to test for an effect of trait type on the MDI value.

### Measurement error

To identify the impact of ME on model fitting, we first compiled estimates of ME for each species and trait value.

For body mass, we obtained the mean and standard deviation following ref. [65]. Specifically, we obtained these values from the source data [76] where possible, calculating a standard deviation from the range of mass values when standard deviations were not reported (following [77]) or using the averaged standard deviation across species when a sample size and mean were the only values reported in ref. [76] or for cases where measurements were only made on a single individual [78] (*n* = 15). We then calculated the ME for each species using the formulae presented in ref. [65].

For plumage pPC axes, the data used in main analyses were calculated from reflectance spectra averaged across individuals, so we first recalculated the spectral indices used in our main analyses on each individual. For both plumage and song pPC axes, we projected individual-level data into pPC scores [54] and directly calculated the ME on these pPC scores (i.e., as |(x_individual_-x_species.mean_)/x_species.mean_|) and then calculated the average ME for each species X trait combination. For downstream analyses of the impact of ME, we removed trait measurements with only one measurement (male plumage *n* = 16, female plumage *n* = 24, song *n* = 37).

For the remaining pPC axes describing resource-use traits, we did not have individual measurements, so we first simulated datasets comprising the same number of measurements as in the empirical dataset by sampling from normal distributions with means and standard deviations of empirical values. We then calculated the pPC scores and ME as above. Likewise, for downstream analyses, we removed trait measurements with only one measurement (*n* = 19).

To test for a potential effect of ME on our results, we computed the median ME for the subset of species and traits corresponding to each of our analyses. We then tested for a relationship between this median ME and the relative index of support for competition models using multiple linear regression analyses. Because increasing rates models may receive inflated support with high ME [48], we also analyzed the effect of ME on the slope parameter of the DD_exp_ model, which was commonly the best-fit model in our analyses.

## Supporting information

S1 Fig Biogeographical stochastic maps constructed in BioGeoBEARS return largely concordant estimates for ancestral ranges over the last 35 My. Plotted is the average proportion of sympatry designations that are identical (i.e., **A**_i,j_[1] = **A**_i,j_[2]) in pairwise comparisons from the root (right) to the tip (left) of the tanager phylogeny across all 50 stochastic maps used in the analyses presented in the main text (Fig 2). Light gray lines represent all pairwise comparisons, and the black line represents the smoothed average value across all pairwise comparisons. My, million years.
(PDF)

S2 Fig Habitat categories for each species, plotted along the tips of the Thraupidae phylogeny. (PDF)

S3 Fig Diet categories for each species, plotted along the tips of the Thraupidae phylogeny. (PDF)

S4 Fig Year-round territorial species, plotted along the tips of the Thraupidae phylogeny. (PDF)

S5 Fig Description of the algorithm used for fitting models incorporating interspecific interactions to subgroups trimmed from the larger phylogeny. (PDF)

S6 Fig MLEs of parameter values for models incorporating interspecific interactions (see S4 Data). (A), MLEs of the strength of competition (*S* values) from the MC model, here plotted as the log-transformed absolute value of *S* for each trait/subset combination. The size of points reflects the relative support for the MC model against models without interactions (i.e., MC_wi_/(max(BM_wi_,OU_wi_)+MC_wi_), where “wi” represents the Akaike weight); points are filled when support for the MC model is greater than support for a noninteraction model. (B), MLEs of the slope parameter (*b* values) from the DD_lin_ models, calculated as above. b > 0 indicates positive DD, while b < 0 indicates negative DD. Note: Nine traits where *b* > 0.01, all of which had very low support, were removed for plotting. (C), MLEs of the slope parameter (*r* values) from the DD_exp_ models, calculated as above. r > 0 indicates positive DD, while r < 0 indicates negative DD. The size of points reflects the relative support for the DD models against models without interactions. BM, Brownian motion; DD, diversity-dependence; DD_exp_, exponential diversity-dependent mode; DD_lin_, linear diversity-dependent model; MC, matching competition, MLE, maximum likelihood estimate; OU, Ornstein-Uhlenbeck.
(PDF)

S7 Fig Model-fitting on a sample of 10 posterior trees yields qualitatively similar results to models fit to the MCC tree (see S4 Data). The relative support for any model that incorporates interspecific interactions (MC, DD_lin_, or DD_exp_), is plotted ± the standard deviation across posterior fits for each trait: (A-D), species grouped by diet, (E-G) species grouped by habitat, and (H) species defending year-round territories. Filled points represent cases where the mean relative support for a model with competition is higher than for a model excluding competition (i.e., relative support > 0.5). Traits (1–27) are defined in S1 Table. DD_exp_, exponential diversity-dependent mode; DD_lin_, linear diversity-dependent model; MC, matching competition; MCC, maximum clade credibility.
(PDF)

S8 Fig ME is positively correlated with the parameter estimate of the slope in the DDexp model. Plotted is the median ME for the subset of species included in the analyses and the estimate of the *r* parameter of the DD_exp_ model. Filled points are those for which the Akaike weight of the DD_exp_ model ≥ 0.5. The vertical line at 0.7 represents the point beyond which support for competition models drops precipitously (see S18 Fig). The relationship between the *r* parameter and median ME is not affected by model support (S6 Table, S1 Data, S4 Data). DD_exp_, exponential diversity-dependent mode; ME, measurement error.
(PDF)

S9 Fig The tempo of ecological and social trait evolution (see S6 Data). (A), Maximum likelihood value of the *σ*^2^ (evolutionary rate) parameter from the BM model fit to standardized trait values show that song traits evolve more rapidly than resource-use or plumage traits in either sex. Plotted are mean ± standard deviation from fits across 100 posterior trees (black points, error bars) and the estimates from the maximum clade credibility tree (open red circles). (B), MDI estimates similarly indicate that song traits accumulate within-clade disparity more rapidly than resource-use or plumage traits in either sex. Plotted are mean ± standard deviation from fits across 100 posterior trees (black points, error bars) and the estimates from the maximum clade credibility tree (open red circles). BM, Brownian motion; MDI, morphological disparity index.
(PDF)

S10 Fig Additional indices of the tempo of ecological and social trait evolution (see S6 Data). (A), Mean of the ln-transformed felsens show that song and plumage traits evolve more rapidly than resource-use traits. Plotted are mean ± standard deviation of mean values for each class of trait from fits across 100 posterior trees. (B), Mean of ln-transformed MLE estimates of the *σ*^2^ (evolutionary rate) parameter from the BM model fit to untransformed trait values, from model fits incorporating ME in geiger, similarly indicate that song and plumage traits evolve more rapidly than resource-use traits. Plotted are mean ± standard deviation of ln-transformed *σ*^2^ values for each class of trait calculated across 100 posterior trees. BM, Brownian motion; ln, log-transformed; ME, measurement error; MLE, maximum likelihood estimate.
(PDF)

S11 Fig Additional indices of the tempo of ecological and social trait evolution (see S6 Data). (A), Mean of the ln-transformed felsens show that song and plumage traits evolve more rapidly than resource-use traits. Plotted are mean ± standard deviation from fits across 100 posterior trees (black points, error bars) and the estimates from the maximum clade credibility tree (open red circles). (B), Mean of ln-transformed MLE estimates of the *σ*^2^ (evolutionary rate) parameter from the BM model fit to untransformed trait values, from model fits incorporating ME, similarly indicate that song and plumage traits evolve more rapidly than resource-use traits. Plotted are mean ± standard deviation from fits across 100 posterior trees (black points, error bars) and the estimates from the maximum clade credibility tree (open red circles). BM, Brownian motion; ln, log-transformed; ME, measurement error; MLE, maximum likelihood estimate.
(PDF)

S12 Fig Frequency distributions of mean differences in rates of trait evolution (MLEs of σ2 from the BM model fit to *z*-transformed data across 100 posterior trees, see S6 Data) between a, ecomorphology and male plumage (Tukey HSD test mean *p* = 0.72, 95% CI [0.42,0.98]); b, ecomorphology and female plumage (Tukey HSD test mean *p* = 0.94, 95% CI [0.75, 1.0]); c, ecomorphology and song (Tukey HSD test mean *p* < 0.001, 95% CI [9e-6, 0.003]); d, male plumage and female plumage (Tukey HSD test mean *p* = 0.94, 95% CI [0.75, 1.0]); e, male plumage and song (Tukey HSD test mean *p* = 0.008, 95% CI [0.001, 0.03]); and f, female plumage and song (Tukey HSD test mean *p* = 0.003, 95% CI [0.001, 0.016]). Rates for song traits are significantly higher than rates for other traits in fits to trait data and 100 posterior trees (all ANOVA significant, mean F_3,23_ = 12.92 [range 5.83–20.91]). BM, Brownian motion; CI, confidence interval; HSD, honest significant difference; MLE, maximum likelihood estimate.
(PDF)

S13 Fig Frequency distributions of mean differences in MDI estimates (see S6 Data) between a, ecomorphology and male plumage (Tukey HSD test mean *p* = 0.15, 95% CI [0.11, 0.23]); b, ecomorphology and female plumage (Tukey HSD test mean *p* = 0.29, 95% CI [0.23, 0.38]); c, ecomorphology and song (Tukey HSD test mean *p* < 0.001, 95% CI [6e-6, 3e-5]); d, male plumage and female plumage (Tukey HSD test mean *p* = 0.98, 95% CI [0.92, 1.0]); e, male plumage and song (Tukey HSD test mean *p* = 0.02, 95% CI [0.009, 0.03]); and f, female plumage and song (Tukey HSD test mean *p* = 0.007, 95% CI [0.002, 0.02]). Rates for song traits are significantly higher than rates for other traits in fits to trait data and 100 posterior trees (all ANOVA significant, mean F_3,23_ = 13.99 [range 12.14–16.01]). BM, Brownian motion; CI, confidence interval; HSD, honest significant difference; MDI, morphological disparity index.
(PDF)

S14 Fig Frequency distributions of mean differences in ln-felsens across 100 posterior trees (see S6 Data) between a, ecomorphology and male plumage (Tukey HSD test mean *p* = 0.006, 95% CI [0.004, 0.009]); b, ecomorphology and female plumage (Tukey HSD test mean *p* = 0.033, 95% CI [0.024, 0.043]); c, ecomorphology and song (Tukey HSD test mean *p* = 0.005, 95% CI [0.003, 0.007]); d, male plumage and female plumage (Tukey HSD test mean *p* = 0.90, 95% CI [0.87, 0.94]); e, male plumage and song (Tukey HSD test mean *p* = 0.94, 95% CI [0.90, 0.98]); and f, female plumage and song (Tukey HSD test mean *p* = 0.99, 95% CI [0.98, 1]). Rates for resource-use traits are lower than rates for other traits in fits to trait data and 100 posterior trees (all ANOVA significant, mean F_3,23_ = 6.49 [range 5.92–7.30]). CI, confidence interval; HSD, honest significant difference; ln, log-transformed.
(PDF)

S15 Fig Frequency distributions of mean differences in rates of trait evolution (MLE of ln(σ2) from the BM model fit to untransformed data with ME incorporated across 100 posterior trees, see S6 Data) between a, ecomorphology and male plumage (Tukey HSD test mean *p* = 0.008, 95% CI [0.005, 0.013]); b, ecomorphology and female plumage (Tukey HSD test mean *p* = 0.03, 95% CI [0.02, 0.046]); c, ecomorphology and song (Tukey HSD test mean *p* = 0.051, 95% CI [0.03, 0.08]); d, male plumage and female plumage (Tukey HSD test mean *p* = 0.95, 95% CI [0.93, 0.97]); e, male plumage and song (Tukey HSD test mean *p* = 0.55, 95% CI [0.48, 0.66]); and f, female plumage and song (Tukey HSD test mean *p* = 0.88, 95% CI [0.83, 0.94]). Rates for resource-use traits are lower than rates for other traits in fits to trait data and 100 posterior trees (all ANOVA significant, mean F_3,23_ = 5.32 [range 4.47–6.22]). BM, Brownian motion; CI, confidence interval; HSD, honest significant difference; ln, log-transformed; ME, measurement error; MLE, maximum likelihood estimate.
(PDF)

S16 Fig Disparity through time plots for each trait show that song traits accumulate within-clade disparity faster than plumage and song traits. Relative time is plotted from the origin of the clade (rel. time = 0.0) to the present (rel. time = 1.0). rel, relative.
(PDF)

S17 Fig Plots of the ln-transformed MLE of *σ*2 (the evolutionary rate parameter) from the BM model plotted against the relative support for a model with competition (see Fig 2 in the main text for more details) for the same analyses presented in Fig 2 (see S4 Data). (A), All data together reveal a negative relationship (effect of ln(*σ*^2^) in logistic regression: *z* = −3.45, *p* < 0.001, model AIC = 199.08), but (B) this relationship is largely driven by differences between trait types in evolutionary rates (effect of ln(*σ*^2^) in logistic regression: *z* = −0.18, *p* = 0.86, model AIC = 186.48). Nevertheless, the trend in the predicted direction is suggestive and an important avenue for future research. AIC, Akaike Information Criterion; BM, Brownian motion; ln, log-transformed; MLE, maximum likelihood estimate.
(PDF)

S18 Fig Relationship between median ME and the relative support for the best model incorporating competition (see S1 Data, S4 Data). ME, measurement error.
(PDF)

S1 Table Description of traits. (DOCX)

S2 Table Loadings for each of the pPC axes presented in S1 Table. Number refers to number of species with trait data (in parentheses, number of species in the final biogeographical analyses with 323 species [see Materials and methods]). For descriptions of variables, see refs [30,31,53]. pPC, phylogenetic principal component.
(DOCX)

S3 Table Modes of trait evolution in tanagers. Best-fit evolutionary models for each trait in each subgrouping were selected as the model with the lowest AICc score in the majority of the 50 fits across incorporating uncertainty in biogeography and partition membership. All analyses were conducted on the MCC tree. Trait numbers correspond to traits in S1 Table. AICc, Akaike Information Criterion; BM, Brownian motion; −DD_exp_, negative exponential diversity-dependent model; +DD_exp_, positive exponential diversity-dependent model; −DD_lin_, negative linear diversity-dependent model; +DD_lin_, positive linear diversity-dependent model; MC, matching competition; MCC, maximum clade credibility; OU, Ornstein-Uhlenbeck.
(DOCX)

S4 Table Results from pairwise regression analyses. If the model term remained significant after the Bonferroni correction, the sign of the model estimate is presented along with information on the simulation scenarios in which the statistical significance occurred (i.e., BM versus OU null simulations as well as the Szymkiewicz-Simpson coefficient threshold used for sympatry [5%, 20%, 50%, 80%, or all four]). Models for which there is evidence for sympatric convergence, even after controlling for similarity in habitat and A, diet (for models fit to members of the same habitat) or B, habitat (for models fit to species with the same diet) or C, both diet and habitat (for models fit to year-round territorial species), are highlighted in bold. BM, Brownian motion; OU, Ornstein-Uhlenbeck.
(DOCX)

S5 Table Multiple linear regression analyses of the effect of trait type on statistical support for models incorporating competition, controlling for the effect of ME. Resource-use traits were set as the reference group so that plumage and song variables test for main effect differences between these traits and resource-use traits. ME, measurement error.
(DOCX)

S6 Table Multiple linear regression analyses of the effect of ME on the MLE of the slope parameter (*r*) of the DDexp model. The effect of this relationship does not depend on the level of statistical support for the DD_exp_ model (i.e., the interaction term is not significant). DD_exp_, exponential diversity-dependent model; ME, measurement error; MLE, maximum likelihood estimate.
(DOCX)

S1 Data Main dataset containing trait data used in analyses. (XLSX)

S2 Data Compressed file with three phylogenies. ‘Tanager_100_posterior_trees.nex’ is a nexus file with 100 trees sampled from the Bayesian posterior distribution, ‘Tanager_MCC_tree.nex’ is a nexus file with the maximum clade credibility tree for all species, and ‘tanagertree_10regions.tree’ is a Newick format tree including only species used in the biogeographical range estimation.
(ZIP)

S3 Data Compressed file with five stochastic maps of biogeography and subgroup membership. ‘diet.simmaps.RData’, ‘habitat.simmaps.RData’, and ‘terr.simmaps.RData’ contain lists in which each element is a stochastic map of diet type, habitat type, and territoriality, respectively. ‘tanagers_RES_ana_events_tables_SMv2.RData’ and ‘tanagers_RES_clado_events_tables_SMv2.RData’ contain stochastic maps of ancestral ranges from BioGeoBEARS.
(ZIP)

S4 Data Excel file containing relative support index (e.g., from Fig 2) and MLEs used in analyses for the MCC tree and posterior trees. MCC, maximum clade credibility; MLE, maximum likelihood estimate.
(XLSX)

S5 Data Compressed file containing.RData files with 100 sets of simulated trajectories of the best-fit models plotted in Fig 3 (i.e., DDexp: Fig 3A, OU: Fig 3B, MC: Fig 3C, BM: Fig 3D). *out*RData files contain individual simulation trajectories (“ivec” identifies the simulation iteration, “dis.sym” refers to the disparity between sympatric lineages at the time indicated, and “disp.allo” refers to the disparity between allopatric lineages at the time indicated), and *sim*RData contains simulated tip values.
(ZIP)

S6 Data Excel file containing estimates of the tempo of trait evolution used in analyses (e.g., presented in Fig 4) for the MCC tree and for posterior trees. MCC, maximum clade credibility. (XLSX)

S1 R Script An example script that fits all five models included in the main analyses to frugivores. (R)

## Abbreviations

AICc: Akaike Information Criterion
BM: Brownian motion
DD: diversity-dependence
DD_exp_: exponential diversity-dependent model
DD_lin_: linear diversity-dependent model
MC: matching competition
MDI: morphological disparity index
ME: measurement error
MLE: maximum likelihood estimate
OU: Ornstein-Uhlenbeck
