# Supplementary figures and images for: Contrasting impacts of competition on ecological and social trait evolution in songbirds
Source: PLoS Biol. 2018 Jan 31;16(1):e2003563. doi: 10.1371/journal.pbio.2003563 (PMC5809094; doi:10.1371/journal.pbio.2003563)

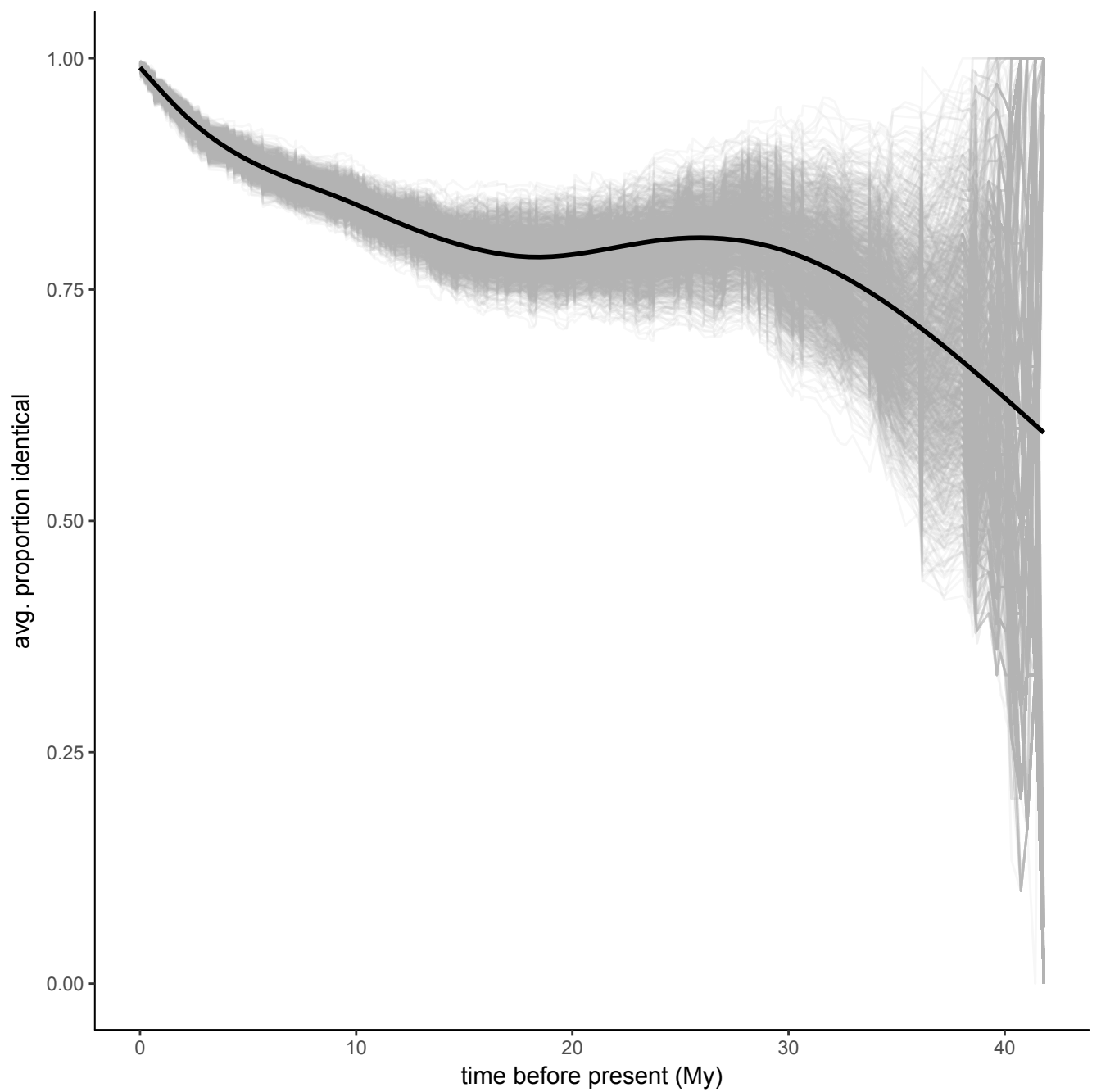

Supplement: S1 Fig — Plotted is the average proportion of sympatry designations that are identical (i.e., Ai,j[1] = Ai,j[2]) in pairwise comparisons from the root (right) to the tip (left) of the tanager phylogeny across all 50 stochastic maps used in the analyses presented in the main text (Fig 2). Light gray lines represent all pairwise comparisons, and the black line represents the smoothed average value across all pairwise comparisons. My, million years. (PDF) [file pbio.2003563.s001.pdf]

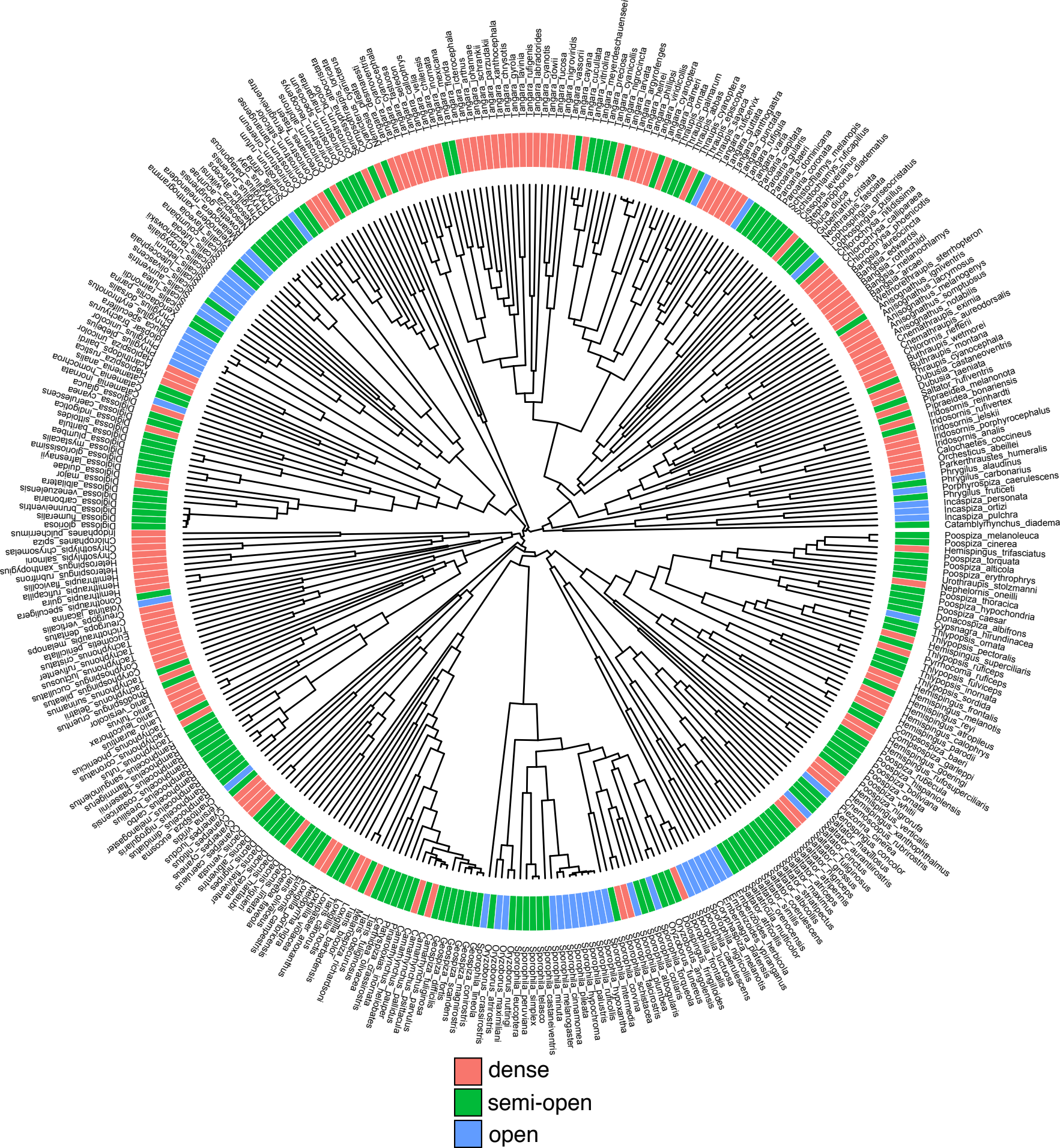

Supplement: S2 Fig — (PDF) [file pbio.2003563.s002.pdf]

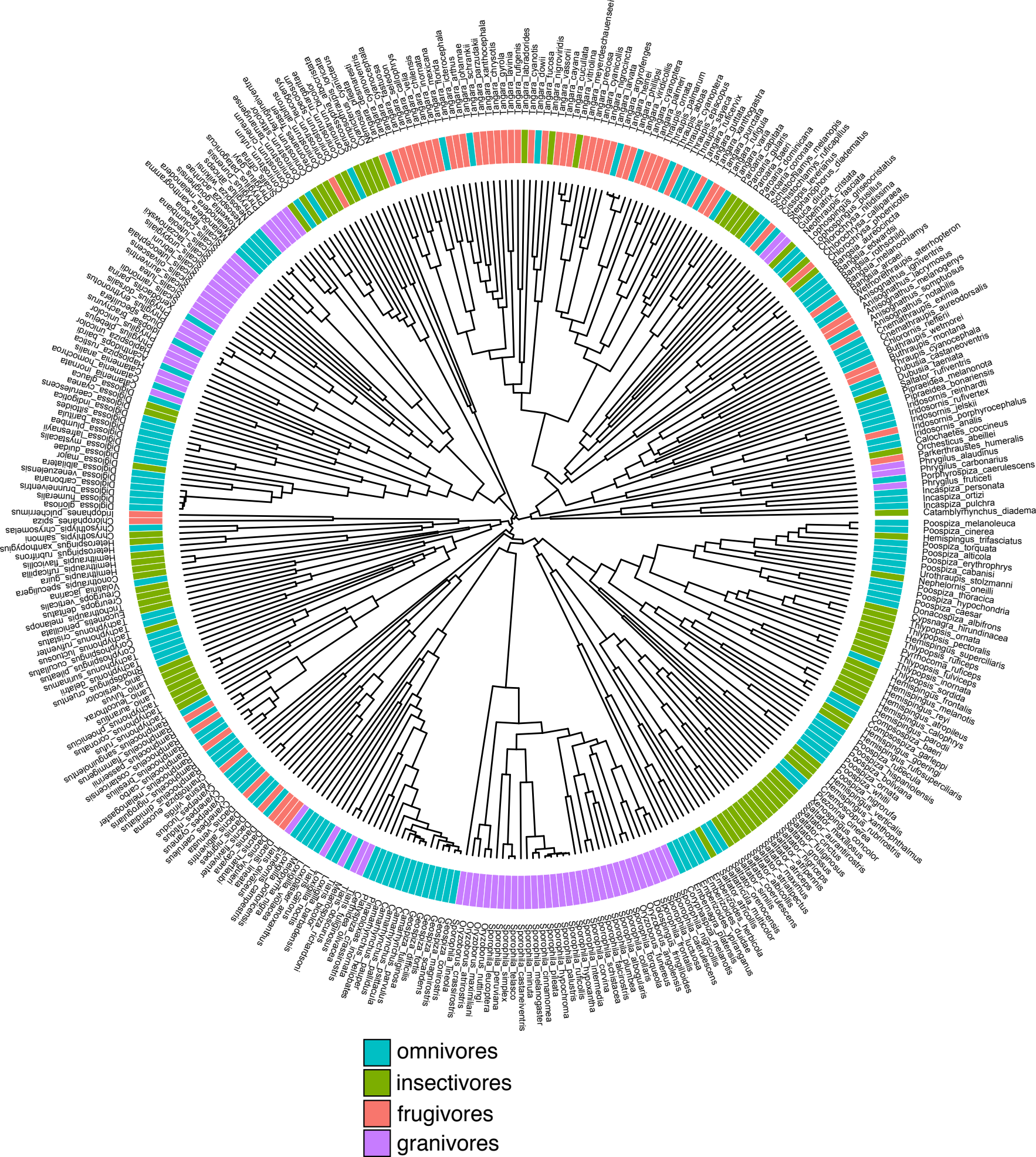

Supplement: S3 Fig — (PDF) [file pbio.2003563.s003.pdf]

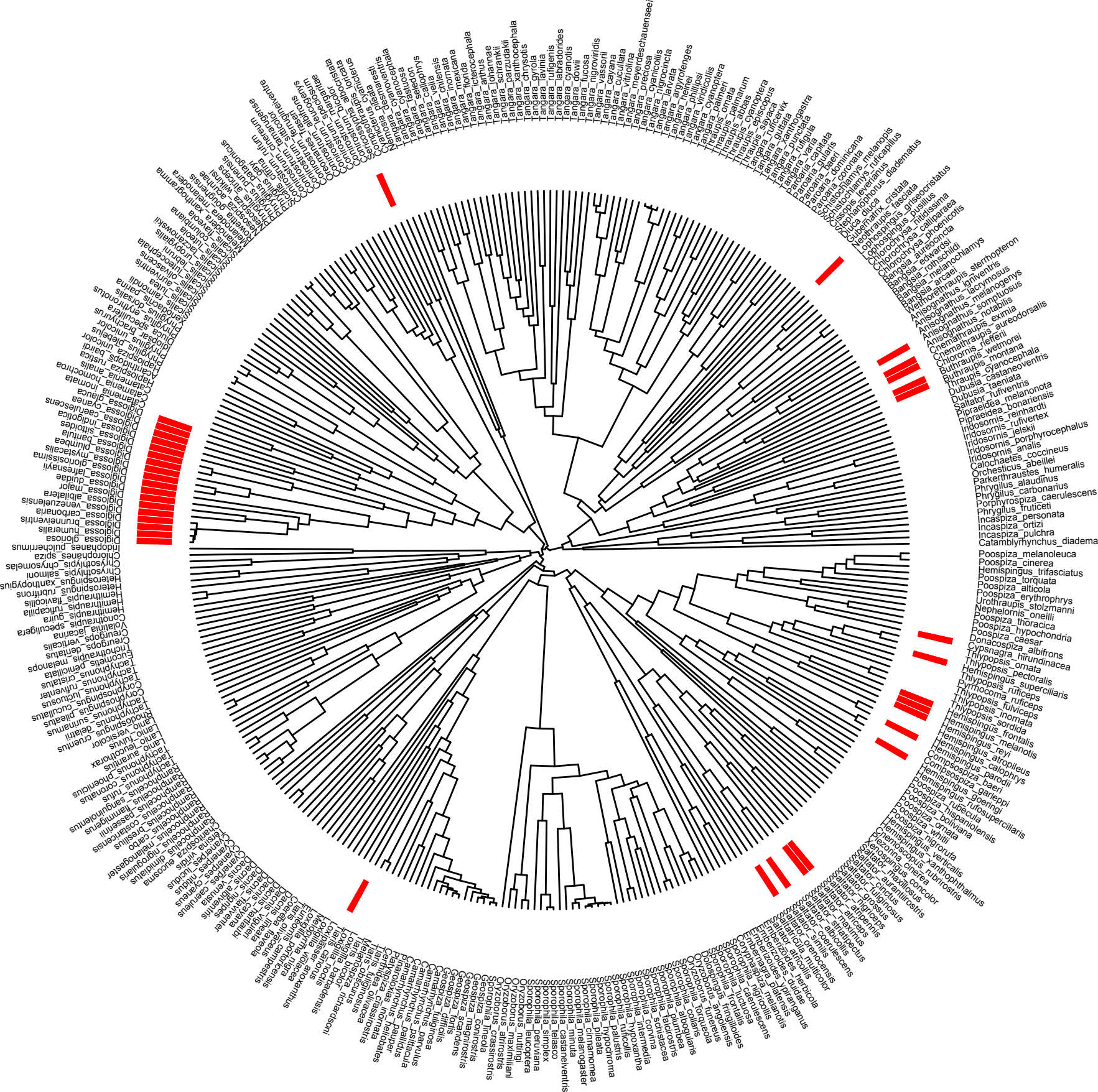

Supplement: S4 Fig — (PDF) [file pbio.2003563.s004.pdf]

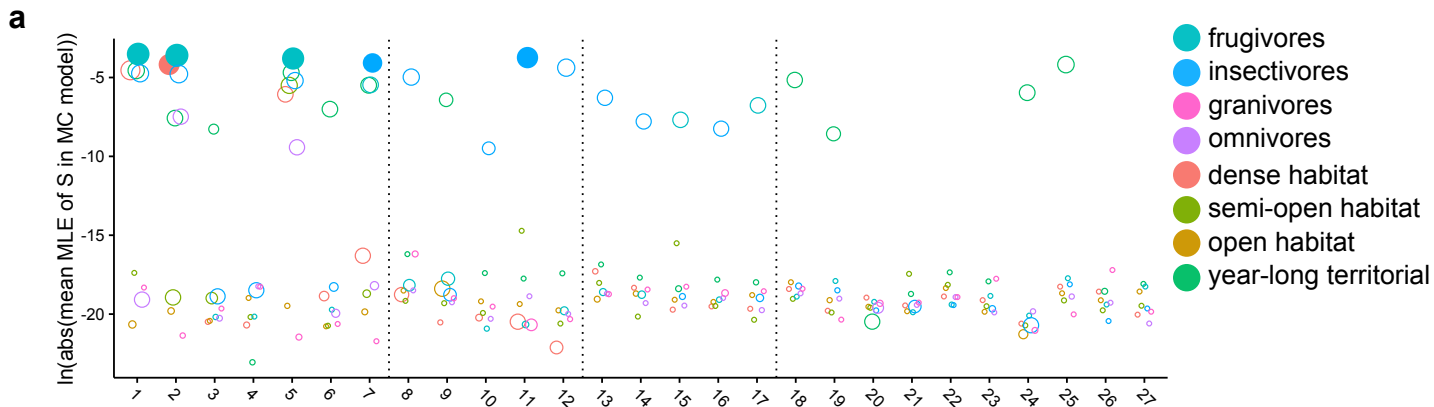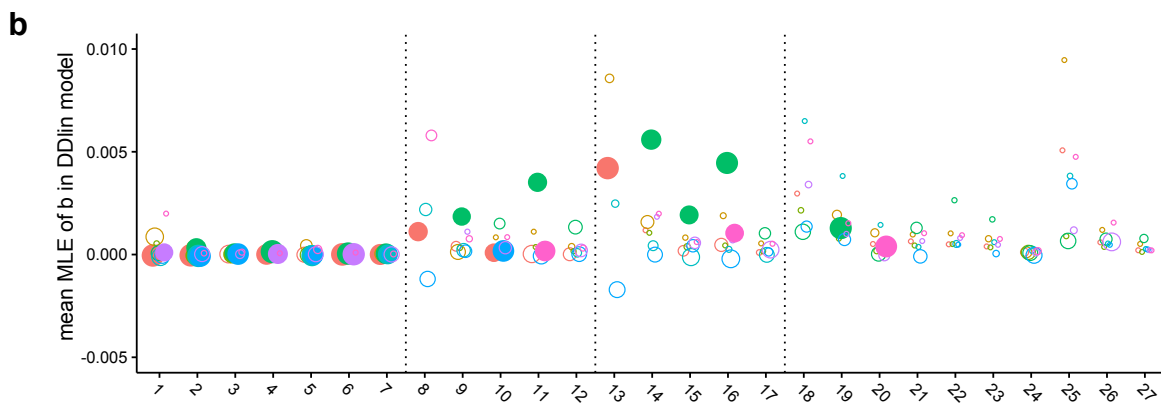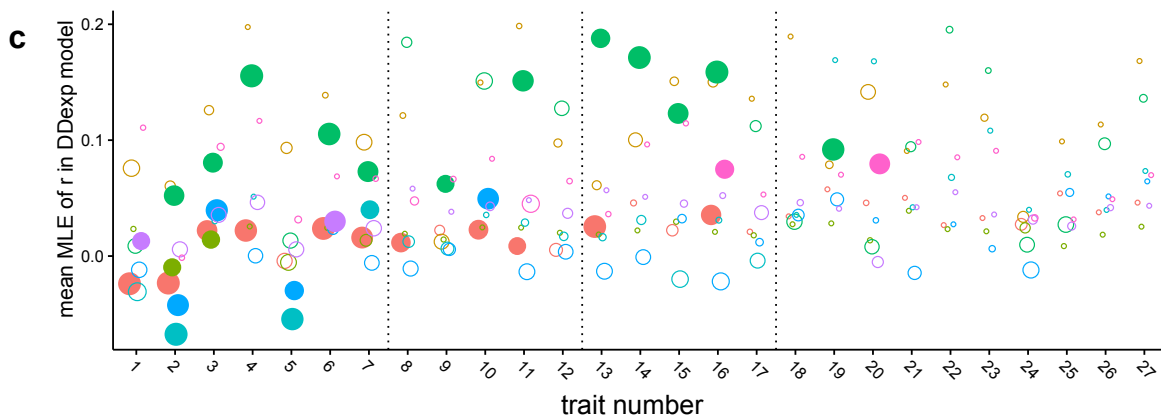

Supplement: S6 Fig — (A), MLEs of the strength of competition (S values) from the MC model, here plotted as the log-transformed absolute value of S for each trait/subset combination. The size of points reflects the relative support for the MC model against models without interactions (i.e., MCwi/(max(BMwi,OUwi)+MCwi), where “wi” represents the Akaike weight); points are filled when support for the MC model is greater than support for a noninteraction model. (B), MLEs of the slope parameter (b values) from the DDlin models, calculated as above. b > 0 indicates positive DD, while b < 0 indicates negative DD. Note: Nine traits where b > 0.01, all of which had very low support, were removed for plotting. (C), MLEs of the slope parameter (r values) from the DDexp models, calculated as above. r > 0 indicates positive DD, while r < 0 indicates negative DD. The size of points reflects the relative support for the DD models against models without interactions. BM, Brownian motion; DD, diversity-dependence; DDexp, exponential diversity-dependent mode; DDlin, linear diversity-dependent model; MC, matching competition, MLE, maximum likelihood estimate; OU, Ornstein-Uhlenbeck. (PDF) [file pbio.2003563.s006.pdf]

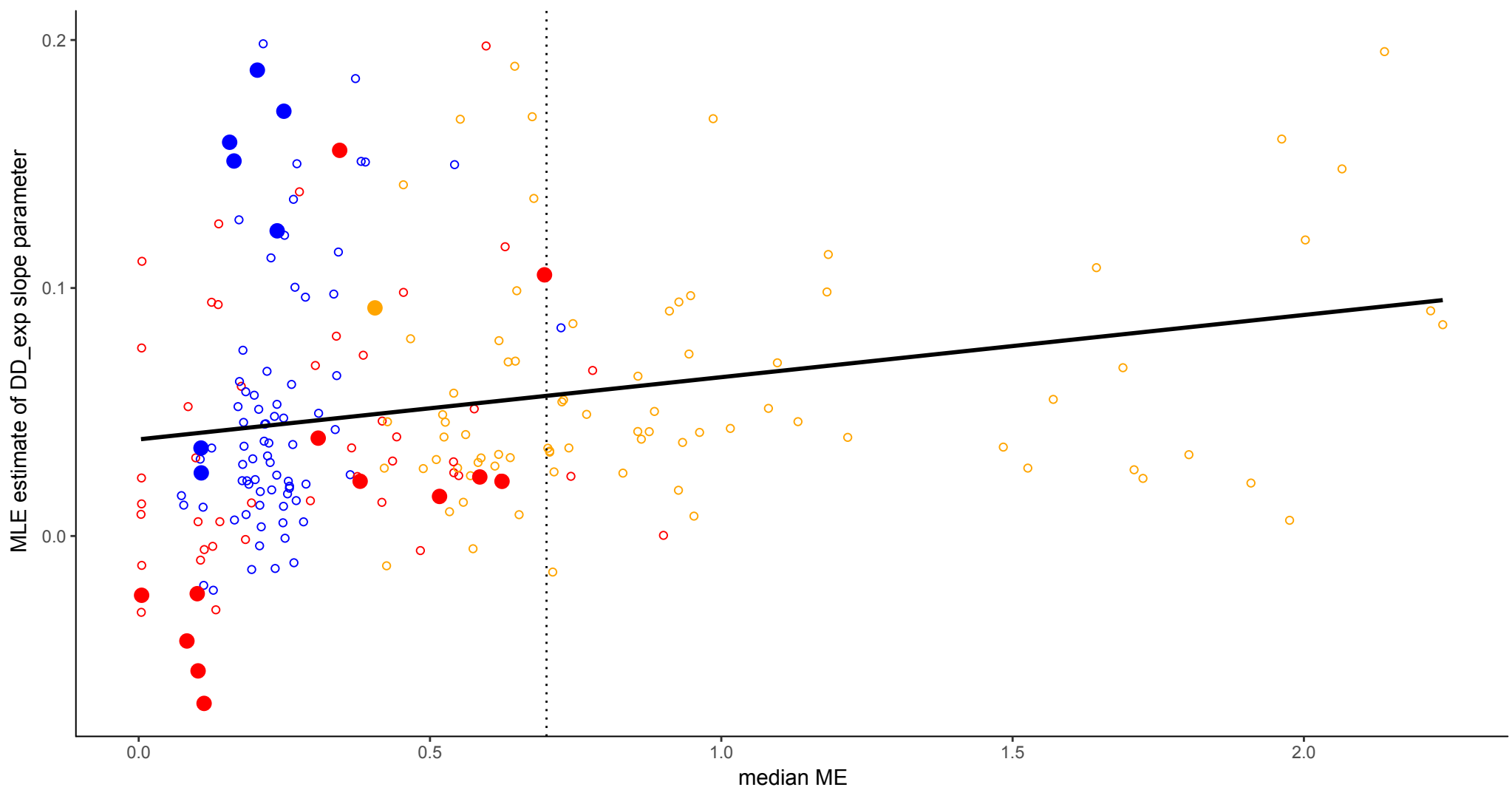

Supplement: S8 Fig — Plotted is the median ME for the subset of species included in the analyses and the estimate of the r parameter of the DDexp model. Filled points are those for which the Akaike weight of the DDexp model ≥ 0.5. The vertical line at 0.7 represents the point beyond which support for competition models drops precipitously (see S18 Fig). The relationship between the r parameter and median ME is not affected by model support (S6 Table, S1 Data, S4 Data). DDexp, exponential diversity-dependent mode; ME, measurement error. (PDF) [file pbio.2003563.s008.pdf]

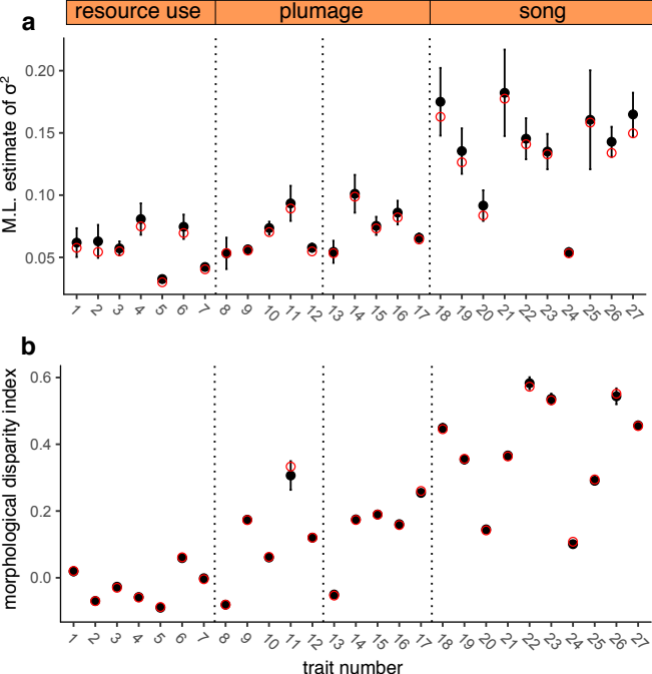

Supplement: S9 Fig — (A), Maximum likelihood value of the σ2 (evolutionary rate) parameter from the BM model fit to standardized trait values show that song traits evolve more rapidly than resource-use or plumage traits in either sex. Plotted are mean ± standard deviation from fits across 100 posterior trees (black points, error bars) and the estimates from the maximum clade credibility tree (open red circles). (B), MDI estimates similarly indicate that song traits accumulate within-clade disparity more rapidly than resource-use or plumage traits in either sex. Plotted are mean ± standard deviation from fits across 100 posterior trees (black points, error bars) and the estimates from the maximum clade credibility tree (open red circles). BM, Brownian motion; MDI, morphological disparity index. (PDF) [file pbio.2003563.s009.pdf]

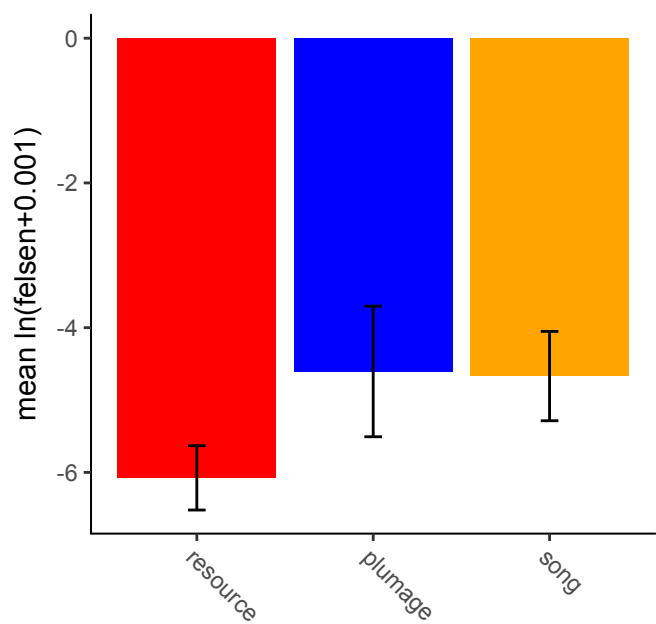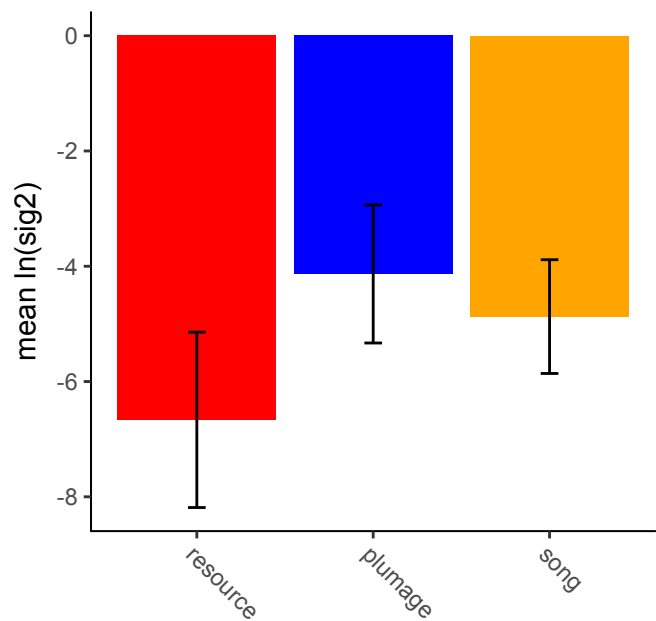

Supplement: S10 Fig — (A), Mean of the ln-transformed felsens show that song and plumage traits evolve more rapidly than resource-use traits. Plotted are mean ± standard deviation of mean values for each class of trait from fits across 100 posterior trees. (B), Mean of ln-transformed MLE estimates of the σ2 (evolutionary rate) parameter from the BM model fit to untransformed trait values, from model fits incorporating ME in geiger, similarly indicate that song and plumage traits evolve more rapidly than resource-use traits. Plotted are mean ± standard deviation of ln-transformed σ2 values for each class of trait calculated across 100 posterior trees. BM, Brownian motion; ln, log-transformed; ME, measurement error; MLE, maximum likelihood estimate. (PDF) [file pbio.2003563.s010.pdf]

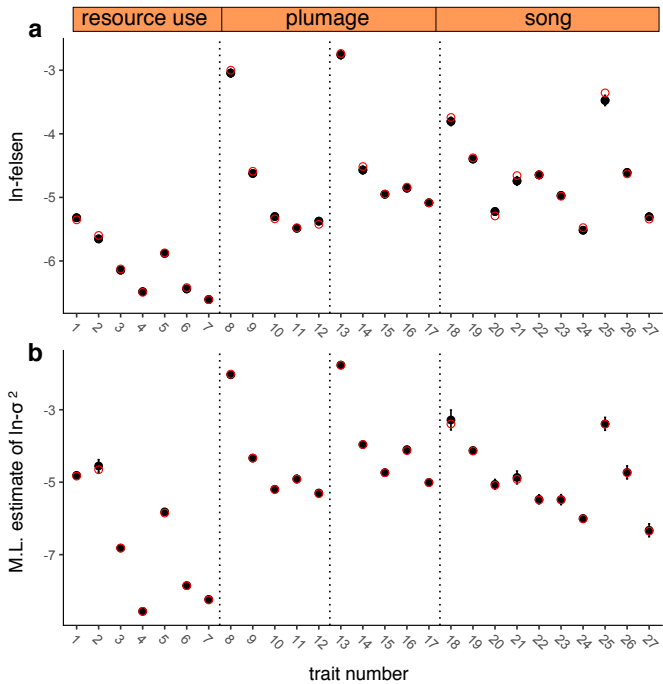

Supplement: S11 Fig — (A), Mean of the ln-transformed felsens show that song and plumage traits evolve more rapidly than resource-use traits. Plotted are mean ± standard deviation from fits across 100 posterior trees (black points, error bars) and the estimates from the maximum clade credibility tree (open red circles). (B), Mean of ln-transformed MLE estimates of the σ2 (evolutionary rate) parameter from the BM model fit to untransformed trait values, from model fits incorporating ME, similarly indicate that song and plumage traits evolve more rapidly than resource-use traits. Plotted are mean ± standard deviation from fits across 100 posterior trees (black points, error bars) and the estimates from the maximum clade credibility tree (open red circles). BM, Brownian motion; ln, log-transformed; ME, measurement error; MLE, maximum likelihood estimate. (PDF) [file pbio.2003563.s011.pdf]

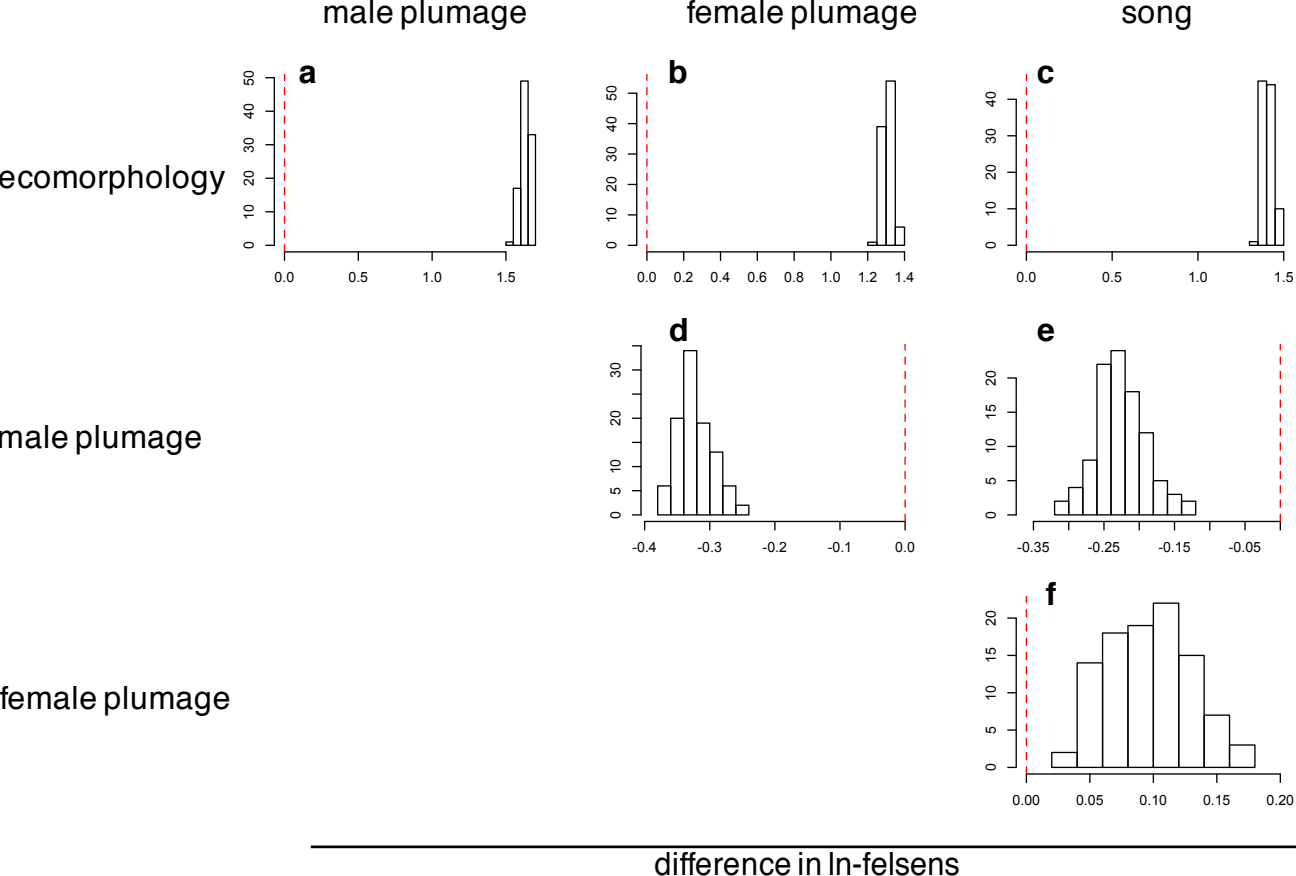

Supplement: S14 Fig — Rates for resource-use traits are lower than rates for other traits in fits to trait data and 100 posterior trees (all ANOVA significant, mean F3,23 = 6.49 [range 5.92–7.30]). CI, confidence interval; HSD, honest significant difference; ln, log-transformed. (PDF) [file pbio.2003563.s014.pdf]

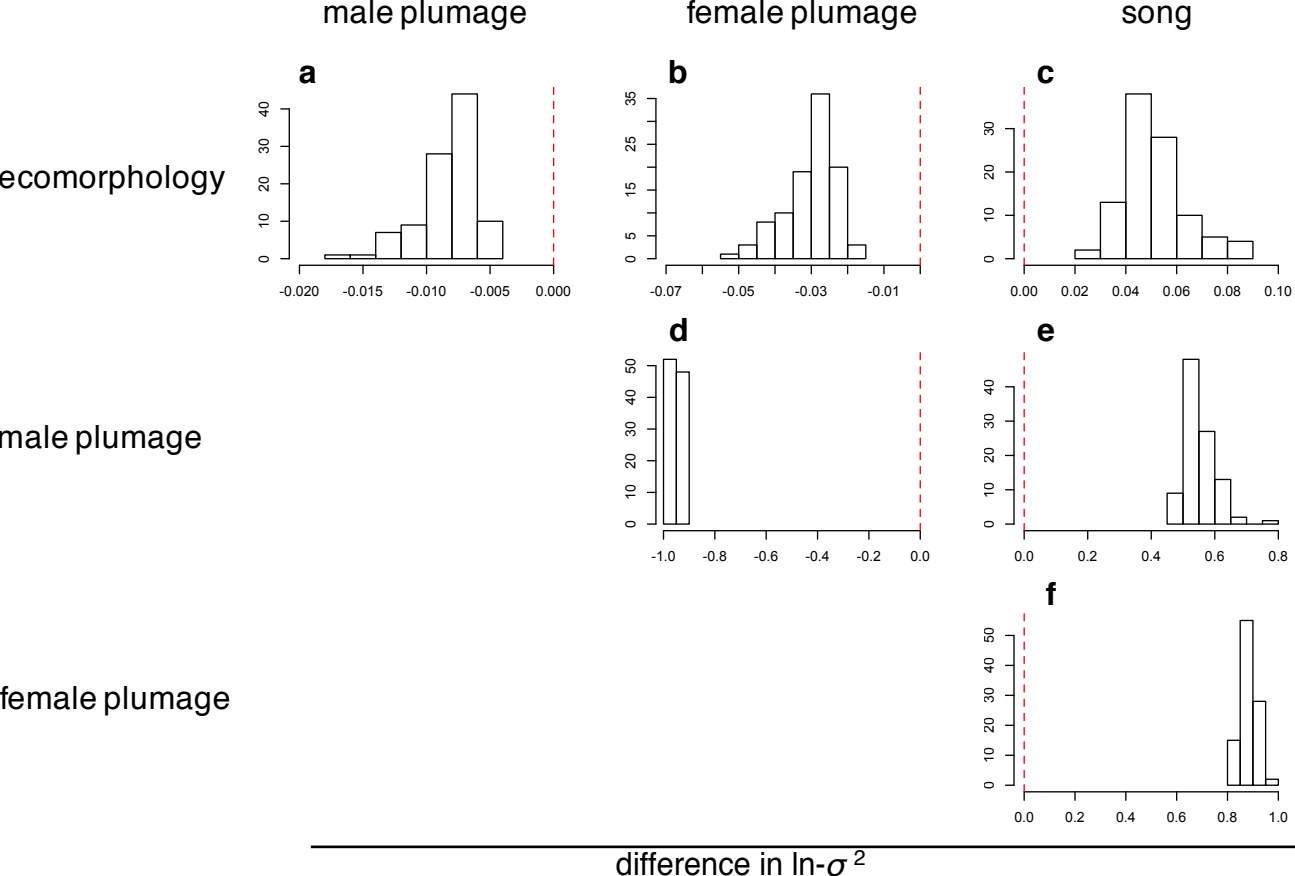

Supplement: S15 Fig — Rates for resource-use traits are lower than rates for other traits in fits to trait data and 100 posterior trees (all ANOVA significant, mean F3,23 = 5.32 [range 4.47–6.22]). BM, Brownian motion; CI, confidence interval; HSD, honest significant difference; ln, log-transformed; ME, measurement error; MLE, maximum likelihood estimate. (PDF) [file pbio.2003563.s015.pdf]

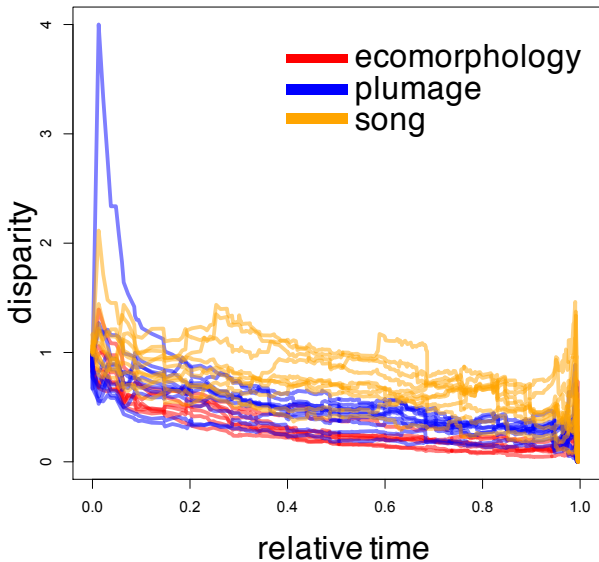

Supplement: S16 Fig — Relative time is plotted from the origin of the clade (rel. time = 0.0) to the present (rel. time = 1.0). rel, relative. (PDF) [file pbio.2003563.s016.pdf]

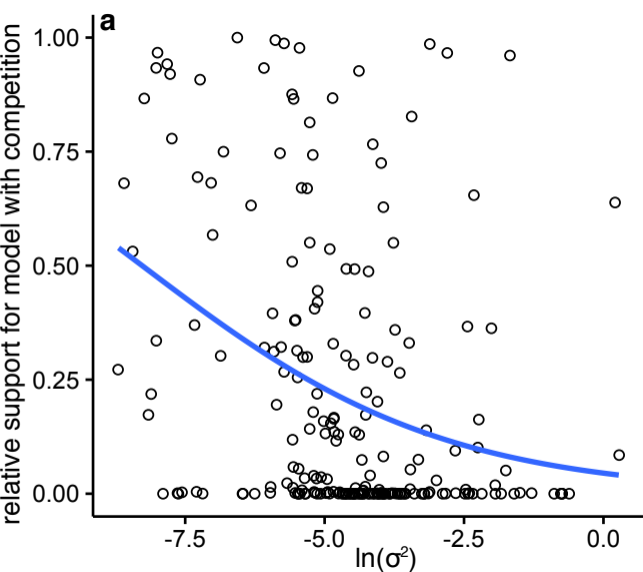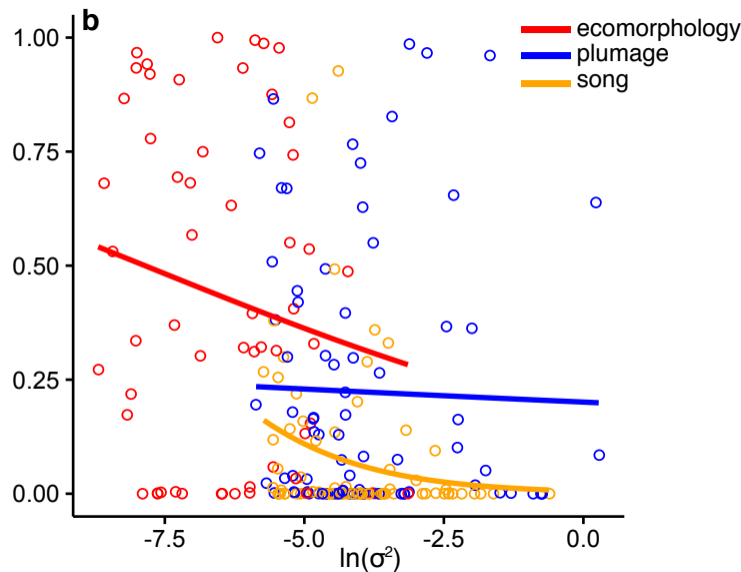

Supplement: S17 Fig — (A), All data together reveal a negative relationship (effect of ln(σ2) in logistic regression: z = −3.45, p < 0.001, model AIC = 199.08), but (B) this relationship is largely driven by differences between trait types in evolutionary rates (effect of ln(σ2) in logistic regression: z = −0.18, p = 0.86, model AIC = 186.48). Nevertheless, the trend in the predicted direction is suggestive and an important avenue for future research. AIC, Akaike Information Criterion; BM, Brownian motion; ln, log-transformed; MLE, maximum likelihood estimate. (PDF) [file pbio.2003563.s017.pdf]

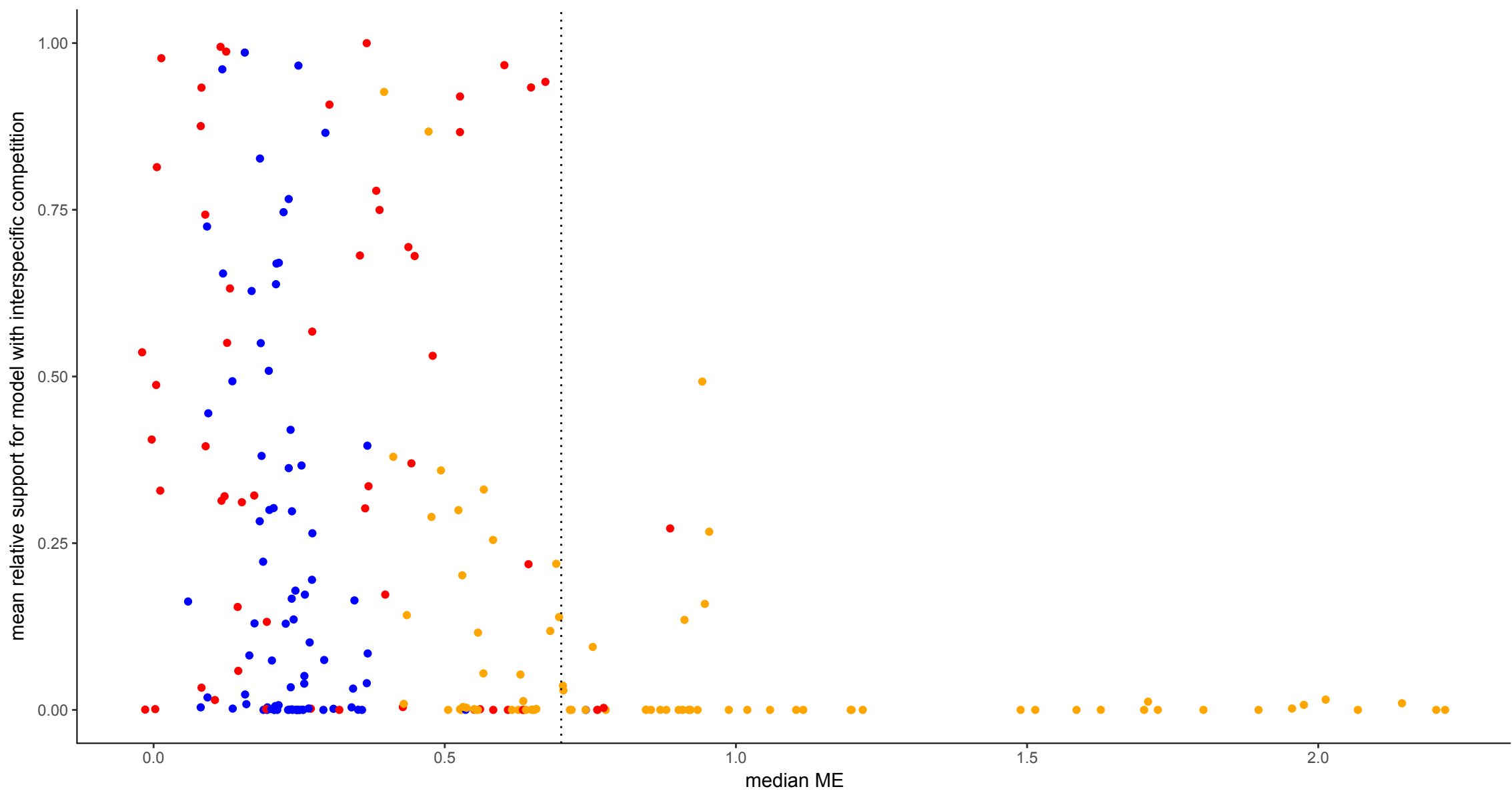

Supplement: S18 Fig — ME, measurement error. (PDF) [file pbio.2003563.s018.pdf]
