# Supplementary material for: Contrasting impacts of competition on ecological and social trait evolution in songbirds
Source: PLoS Biol. 2018 Jan 31;16(1):e2003563. doi: 10.1371/journal.pbio.2003563 (PMC5809094; doi:10.1371/journal.pbio.2003563)
Supplement: S5 Fig — (PDF) [file pbio.2003563.s005.pdf]

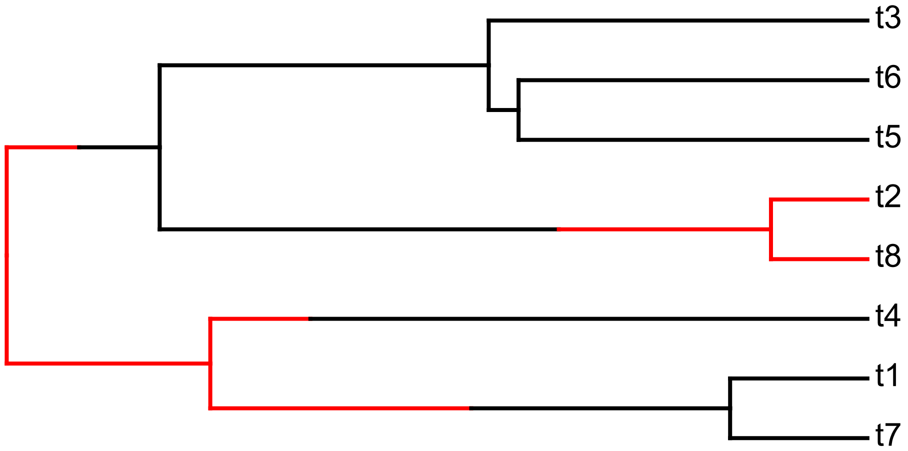


**Steps for fitting MC & DD models to a sub-group identified by a stochastic map (e.g., diet category):**

*In this example, corresponding to the figure above, the black branches are the ones we want to preserve.*

1. Trim all branches that do not lead to any descendants in focal category and which can be removed without losing any part of the history of the focal category (t2 dropped at this step).
2. Trim stochastic map of biogeography to contain only branches remaining after step 1. Species can only compete if they both occupy the same biogeographic region and belong to the same subcategory.
3. Calculate variance-covariance matrix; treating non-focal guild branches as evolving via BM (i.e., not influencing trait evolution in other lineages in the focal category)
4. Remove rows and columns from VCV matrix that belong to non-guild members (t8 variance and covariance values removed at this step)
5. Calculate likelihood using data from the sub-group lineages and this VCV matrix (see Harmon et al. 2010).
