# Supplementary material for: Contrasting impacts of competition on ecological and social trait evolution in songbirds
Source: PLoS Biol. 2018 Jan 31;16(1):e2003563. doi: 10.1371/journal.pbio.2003563 (PMC5809094; doi:10.1371/journal.pbio.2003563)
Supplement: S7 Fig — The relative support for any model that incorporates interspecific interactions (MC, DDlin, or DDexp), is plotted ± the standard deviation across posterior fits for each trait: (A-D), species grouped by diet, (E-G) species grouped by habitat, and (H) species defending year-round territories. Filled points represent cases where the mean relative support for a model with competition is higher than for a model excluding competition (i.e., relative support > 0.5). Traits (1–27) are defined in S1 Table. DDexp, exponential diversity-dependent mode; DDlin, linear diversity-dependent model; MC, matching competition; MCC, maximum clade credibility. (PDF) [file pbio.2003563.s007.pdf]

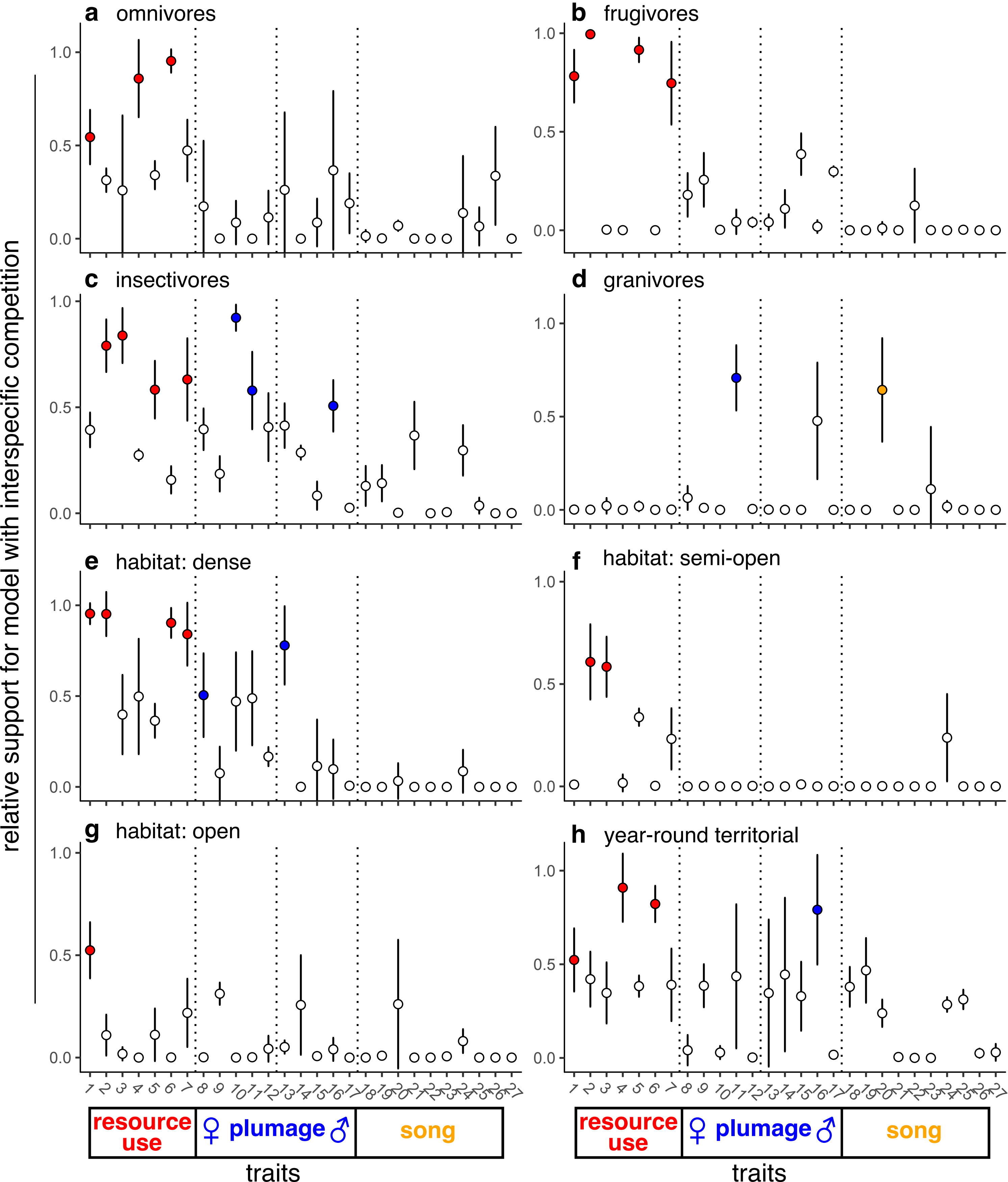

a omnivores
b frugivores
c insectivores
d granivores
e habitat: dense
f habitat: semi-open
g habitat: open
h year-round territorial
relative support for model with interspecific competition
0.0
0.5
1.0
1 2 3 4 5 6 7 8 9 10 11 12 13 14 15 16 17 18 19 20 21 22 23 24 25 26 27
resource use
♀ plumage ♂
song
traits
