# Supplementary material for: Contrasting impacts of competition on ecological and social trait evolution in songbirds
Source: PLoS Biol. 2018 Jan 31;16(1):e2003563. doi: 10.1371/journal.pbio.2003563 (PMC5809094; doi:10.1371/journal.pbio.2003563)
Supplement: S12 Fig — Rates for song traits are significantly higher than rates for other traits in fits to trait data and 100 posterior trees (all ANOVA significant, mean F3,23 = 12.92 [range 5.83–20.91]). BM, Brownian motion; CI, confidence interval; HSD, honest significant difference; MLE, maximum likelihood estimate. (PDF) [file pbio.2003563.s012.pdf]

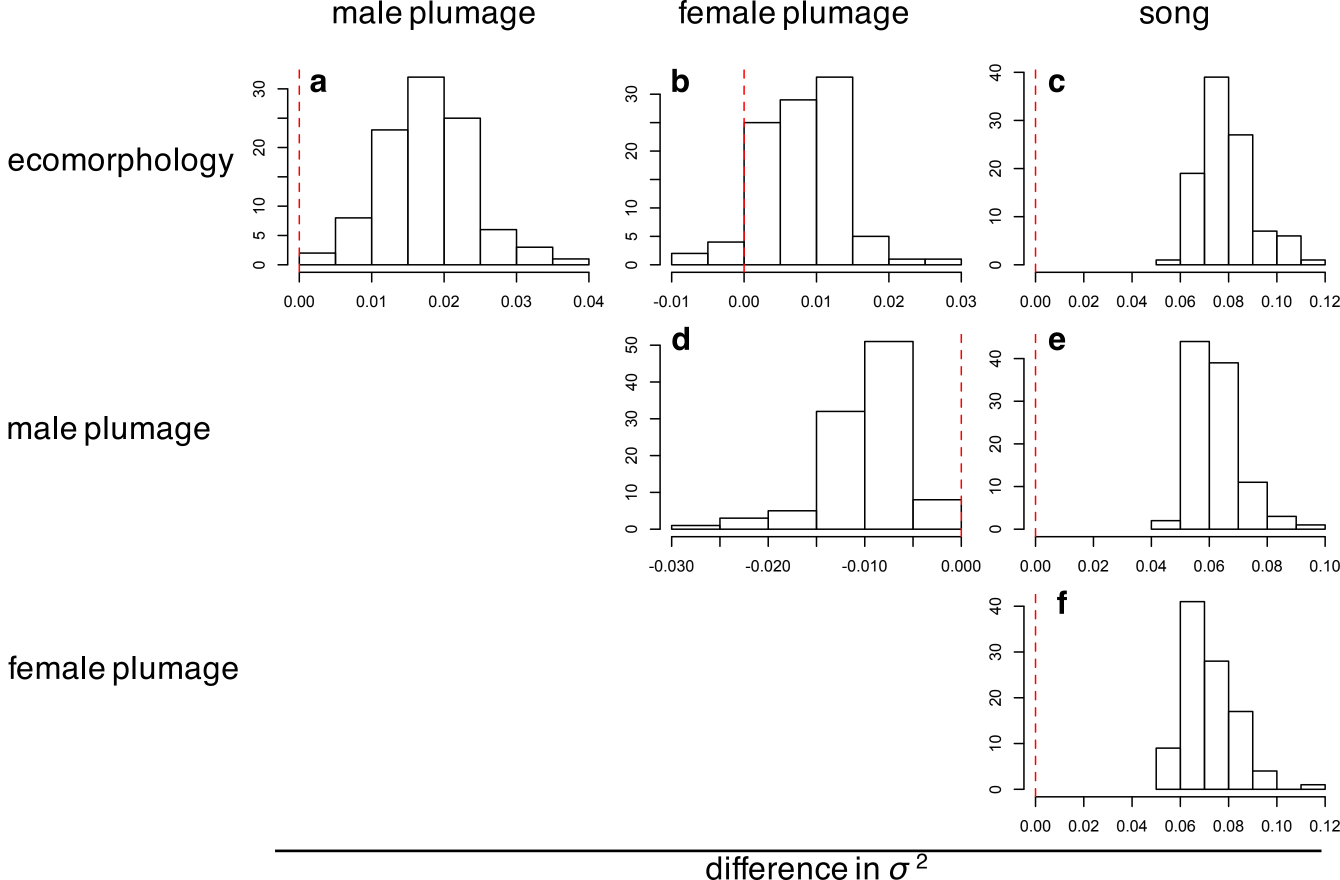
male plumage
female plumage
song
ecomorphology
male plumage
female plumage
a
b
c
d
e
f
difference in σ 2
