# Supplementary material for: Contrasting impacts of competition on ecological and social trait evolution in songbirds
Source: PLoS Biol. 2018 Jan 31;16(1):e2003563. doi: 10.1371/journal.pbio.2003563 (PMC5809094; doi:10.1371/journal.pbio.2003563)
Supplement: S13 Fig — Rates for song traits are significantly higher than rates for other traits in fits to trait data and 100 posterior trees (all ANOVA significant, mean F3,23 = 13.99 [range 12.14–16.01]). BM, Brownian motion; CI, confidence interval; HSD, honest significant difference; MDI, morphological disparity index. (PDF) [file pbio.2003563.s013.pdf]

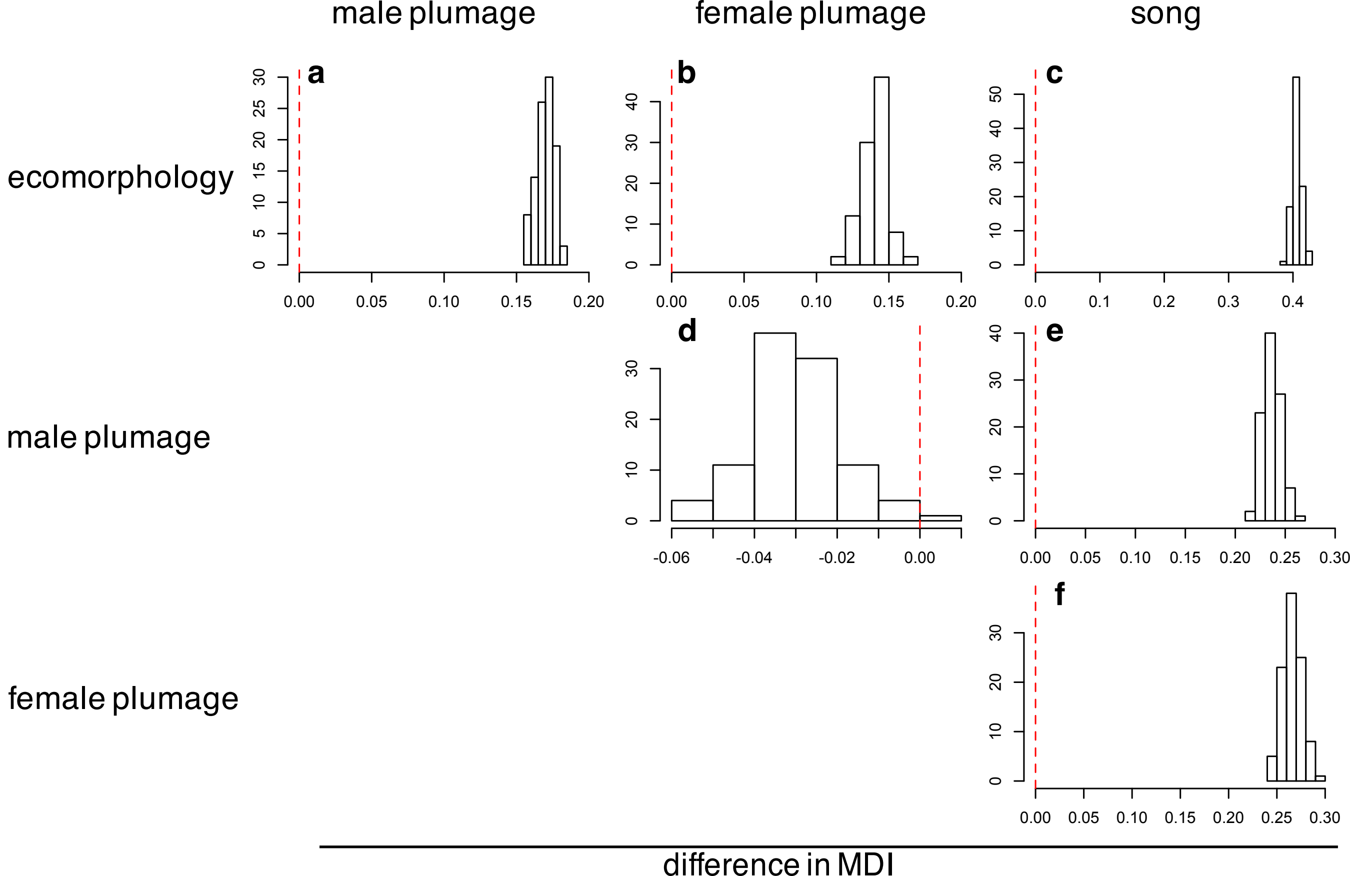

male plumage
female plumage
song
ecomorphology
male plumage
female plumage
a
b
c
d
e
f
difference in MDI
