# Supplementary material for: Contrasting impacts of competition on ecological and social trait evolution in songbirds
Source: PLoS Biol. 2018 Jan 31;16(1):e2003563. doi: 10.1371/journal.pbio.2003563 (PMC5809094; doi:10.1371/journal.pbio.2003563)
Supplement: S1 Table — (DOCX) [file pbio.2003563.s019.docx]

**S1 Table.** Description of traits.

| **#** | **trait ID** | **pPC variance**  **explained** | **description** |
| --- | --- | --- | --- |
| 1 | Body mass | — | ln-transformed body mass |
| 2 | Beak pPC1 | 81.4% | Overall beak size |
| 3 | Beak pPC2 | 13.5% | High scores = shape of beak is relatively long and thin (highest score is *Cyanerpes lucidus*; lowest is *Sporophila cinnamomea*) |
| 4 | Beak pPC3 | 5.2% | High scores = shape of beak is relatively narrow (highest score is *Diglossa carbonaria*; lowest is *Tersina viridis*) |
| 5 | Locomotion pPC1 | 75.5% | Overall size |
| 6 | Locomotion pPC2 | 16.8% | High scores = relatively long tail (highest score is *Donacospiza albifrons*; lowest is *Geospiza magnirostris*) |
| 7 | Locomotion pPC3 | 7.7% | High scores = relatively long wings and short tarsi (highest is *Sericossypha albocristata*; lowest is *Hemispingus goeringi*) |
| 8 | Female Color Disparity pPC1 | 89.7% | High scores = very homogenous patches (highest = *Diglossa* *humeralis*; lowest = *Tangara velia*) |
| 9 | Female Color Disparity pPC2 | 6.9% | High scores = relatively large hue disparity/ span for a particular color volume (NB: low scores tend to be brown and high scores black) (highest = *Compsothraupis loricata*; lowest = *Oryzoborus nuttingi*) |
| 10 | Female Color Disparity pPC3 | 3.4% | High scores = dissimilarity between chroma of patches yet different patches have similar hues (highest = *Ramphocelus sanguinolentus*; lowest = *Thraupis ornata*) |
| 11 | Female Coloration pPC1 | 65.0% | High scores = overall bright, chromatic plumage (highest = *Tangara parzudakii*; lowest = *Compsothraupis loricata*) |
| 12 | Female Coloration pPC2 | 35.0% | High scores = overall chromatic plumage with low reflectiveness (highest = *Ramphocelus sanguinolentus*; lowest = *Thraupis episcopus*) |
| 13 | Male Color Disparity pPC1 | 85.6% | High scores = very homogenous patches (highest = *Tachyphonus luctuosus*; lowest = *Tangara velia*) |
| 14 | Male Color Disparity pPC2 | 10.7% | High scores = relatively large hue disparity/ span for a particular color volume (NB: low scores tend to be brown and high scores black) (highest = *Tachyphonus phoenicius*; lowest = *Orchesticus abeillei*) |
| 15 | Male Color Disparity pPC3 | 3.7% | High scores = dissimilarity between chroma of patches yet different patches have similar hues (highest = *Ramphocelus nigrogularis*; lowest = *Saltator grossus*) |
| 16 | Male Coloration pPC1 | 74.7% | High scores = overall bright, chromatic plumage (highest = *Chlorochrysa phoenicotis*; lowest = *Tachyphonus luctuosus*) |
| 17 | Male Coloration pPC2 | 25.3% | High scores = overall chromatic plumage with low reflectiveness (highest = *Ramphocelus nigrogularis*; lowest = *Thraupis episcopus*) |
| 18 | Song Tempo pPC1 | 74.1% | High scores = long songs (highest = *Ramphocelus bresilius*; lowest = *Coryphaspiza melanotis*) |
| 19 | Song Tempo pPC2 | 19.0% | High scores = songs with few, relatively long, spaced out notes; low scores staccato, many short notes closely spaced (highest = *Thraupis abbas*; lowest = *Diglossa gloriosa*) |
| 20 | Song Tempo pPC3 | 6.9% | High scores = songs with relatively more notes and longer pauses [more notes in a smaller space] (highest = *Thlypopsis fulviceps*; lowest = *Thraupis abbas*) |
| 21 | Whole Song Frequency pPC1 | 42.1% | High scores = low overall frequency range (highest = *Sporophila leucoptera*; lowest = *Cyanerpes lucidus*) |
| 22 | Whole Song Frequency pPC2 | 27.1% | High scores = high amplitude slope (songs get louder) (highest = *Tangara xanthocephala*; lowest = *Iridosornis jelskii*) |
| 23 | Whole Song Frequency pPC3 | 23% | High scores = songs with relatively lower frequency slope and peak frequency; songs that decrease in frequency also increase in amplitude (highest = *Phrygilus erythronotus*; lowest = *Iridosornis rufivertex*) |
| 24 | Whole Song Frequency pPC4 | 7.9% | High scores = higher peak frequency (highest = *Tangara varia*; lowest = *Cypsnagra hirundinacea*) |
| 25 | Note Frequency pPC1 | 86.6% | High scores = fewer noteworthy frequency shifts, overall low range in notes (highest = *Saltator albicollis*; lowest = *Dacnis hartlaubi*) |
| 26 | Note Frequency pPC2 | 10.1% | High scores = songs with few, but large, frequency shifts (highest = *Cyanerpes lucidus*; lowest = *Bangsia aureocincta*) |
| 27 | Note Frequency pPC3 | 3.3% | High scores = tradeoff representing lower average range of notes with higher maximum frequency shift [disparity in within note shifts?] (highest = *Cnemathraupis aureodorsalis*; lowest = *Hemispingus calophrys*) |
