# Supplementary material for: Contrasting impacts of competition on ecological and social trait evolution in songbirds
Source: PLoS Biol. 2018 Jan 31;16(1):e2003563. doi: 10.1371/journal.pbio.2003563 (PMC5809094; doi:10.1371/journal.pbio.2003563)
Supplement: S2 Table — Number refers to number of species with trait data (in parentheses, number of species in the final biogeographical analyses with 323 species [see Materials and methods]). For descriptions of variables, see refs [30,31,53]. pPC, phylogenetic principal component. (DOCX) [file pbio.2003563.s020.docx]

**S2 Table.** Loadings for each of the pPC axes presented in Table 1. Number refers to number of species with trait data (in parentheses, number of species in the final biogeographical analyses with 323 species [see Materials & Methods]). For descriptions of variables, see refs [29,30,50].

| **analysis** | **variable** | **pPC1** | **pPC2** | **pPC3** | **pPC4** |
| --- | --- | --- | --- | --- | --- |
| Beak | Culmen length | -0.74 | +0.68 | +0.02 |  |
| *n* = 350 (319) | Beak width | -0.95 | -0.15 | -0.29 |  |
|  | Beak depth | -0.95 | -0.19 | +0.24 |  |
| Locomotion | Wing | -0.79 | -0.46 | +0.41 |  |
| *n* = 350 (319) | Tarsus | -0.81 | -0.44 | -0.39 |  |
|  | Tail | -0.93 | +0.37 | +0.01 |  |
| Color disparity | Avg. span (f/m) | -0.86 / -0.74 | +0.18 / +0.51 | +0.47 / +0.44 |  |
| *n_f_* = 342 (314); *n_m_* = 341 (312) | Volume (f/m) | -0.99 / -0.99 | -0.09 / -0.10 | -0.05 / -0.03 |  |
|  | Avg hue disparity (f/m) | -0.63 / -0.40 | +0.75 / +0.84 | -0.18 / -0.37 |  |
| Coloration | Avg. brilliance (f/m) | +0.76 / +0.59 | -0.64 / -0.81 |  |  |
| *n_f_* = 342 (314); *n_m_* = 341 (312) | Avg. chroma (f/m) | +0.84 / +0.97 | +0.54 / +0.24 |  |  |
| Song tempo | Song length | +0.98 | -0.21 | -0.02 |  |
| *n* = 321 (294) | Note percentage | -0.47 | -0.36 | -0.81 |  |
|  | Note rate | -0.60 | -0.78 | +0.16 |  |
| Whole song frequency | Peak frequency | -0.30 | -0.08 | -0.20 | +0.93 |
| *n* = 321 (294) | Song frequency range | -0.95 | +0.003 | -0.31 | -0.08 |
|  | Frequency slope | +0.52 | +0.09 | -0.85 | -0.03 |
|  | Amplitude slope | -0.05 | +1.00 | +0.07 | +0.03 |
| Note frequency | Avg note freq. range | -0.68 | +0.62 | -0.39 |  |
| *n* = 313 (287) | Frequency shift rate | -0.99 | -0.13 | -0.0003 |  |
|  | Max frequency shift | -0.69 | +0.62 | +0.37 |  |
