# Supplementary material for: Contrasting impacts of competition on ecological and social trait evolution in songbirds
Source: PLoS Biol. 2018 Jan 31;16(1):e2003563. doi: 10.1371/journal.pbio.2003563 (PMC5809094; doi:10.1371/journal.pbio.2003563)
Supplement: S3 Table — Best-fit evolutionary models for each trait in each subgrouping were selected as the model with the lowest AICc score in the majority of the 50 fits across incorporating uncertainty in biogeography and partition membership. All analyses were conducted on the MCC tree. Trait numbers correspond to traits in S1 Table. AICc, Akaike Information Criterion; BM, Brownian motion; −DDexp, negative exponential diversity-dependent model; +DDexp, positive exponential diversity-dependent model; −DDlin, negative linear diversity-dependent model; +DDlin, positive linear diversity-dependent model; MC, matching competition; MCC, maximum clade credibility; OU, Ornstein-Uhlenbeck. (DOCX) [file pbio.2003563.s021.docx]

**S3 Table. Modes of trait evolution in tanagers.** Best-fit evolutionary models (BM = Brownian motion, OU = Ornstein-Uhlenbeck, MC = matching competition, +DD_exp_ = positive diversity dependent exponential, +DD_lin_ = positive diversity dependent linear, -DD_exp_ = negative diversity dependent exponential, -DD_lin_ = negative diversity dependent linear) for each trait in each subgrouping were selected as the model with the lowest AICc score in the majority of the 50 fits across incorporating uncertainty in biogeography and partition membership. All analyses were conducted on the MCC tree. Trait numbers correspond to traits in S1 Table.

| **trait** | | **partition** | | | | | | | | | | | |
| --- | --- | --- | --- | --- | --- | --- | --- | --- | --- | --- | --- | --- | --- |
|  |  | year-round |  | habitat | | |  | | diet | | | | |
|  |  | territoriality |  | dense | semi-open | open | |  | | fruit | invertebrates | omnivores | seed |
| **resource-use** | 1 | BM |  | -DD_exp_ | OU | OU | |  | | MC | BM | +DD_lin_ | OU |
|  | 2 | +DD_exp_ |  | -DD_exp_ | BM | OU | |  | | -DD_exp_ | -DD_exp_ | BM | OU |
|  | 3 | +DD_exp_ |  | +DD_exp_ | +DD_lin_ | OU | |  | | OU | +DD_exp_ | OU | OU |
|  | 4 | +DD_exp_ |  | +DD_exp_ | OU | OU | |  | | OU | BM | +DD_lin_ | OU |
|  | 5 | BM |  | BM | BM | OU | |  | | -DD_exp_ | -DD_exp_ | BM | OU |
|  | 6 | +DD_exp_ |  | +DD_exp_ | OU | OU | |  | | OU | OU | +DD_lin_ | OU |
|  | 7 | +DD_exp_ |  | +DD_exp_ | OU | OU | |  | | +DD_lin_ | MC | OU | OU |
|  |  |  |  |  |  |  | |  | |  |  |  |  |
|  | 8 | OU |  | +DD_exp_ | OU | OU | |  | | OU | BM | OU | OU |
|  | 9 | +DD_lin_ |  | OU | OU | BM | |  | | OU | OU | OU | OU |
|  | 10 | OU |  | +DD_exp_ | OU | OU | |  | | OU | +DD_lin_ | OU | OU |
| **plumage** | 11 | +DD_exp_ |  | BM | OU | OU | |  | | OU | MC | OU | +DD_lin_ |
|  | 12 | OU |  | OU | OU | OU | |  | | OU | BM | OU | OU |
|  | 13 | +DD_exp_ |  | +DD_exp_ | OU | OU | |  | | OU | BM | OU | OU |
|  | 14 | +DD_exp_ |  | OU | OU | OU | |  | | OU | BM | OU | OU |
|  | 15 | +DD_exp_ |  | OU | OU | OU | |  | | BM | OU | OU | OU |
|  | 16 | +DD_exp_ |  | +DD_exp_ | OU | OU | |  | | OU | -DD_lin_ | OU | +DD_exp_ |
|  | 17 | OU |  | OU | OU | OU | |  | | BM | OU | OU | OU |
|  |  |  |  |  |  |  | |  | |  |  |  |  |
|  | 18 | OU |  | OU | OU | OU | |  | | OU | OU | OU | OU |
|  | 19 | +DD_exp_ |  | OU | OU | OU | |  | | OU | OU | OU | OU |
|  | 20 | OU |  | OU | OU | OU | |  | | OU | OU | OU | +DD_lin_ |
| **song** | 21 | OU |  | OU | OU | OU | |  | | OU | OU | OU | OU |
|  | 22 | OU |  | OU | OU | OU | |  | | OU | OU | OU | OU |
|  | 23 | OU |  | OU | OU | OU | |  | | OU | OU | OU | OU |
|  | 24 | BM |  | OU | OU | OU | |  | | OU | BM | OU | OU |
|  | 25 | BM |  | OU | OU | OU | |  | | OU | OU | OU | OU |
|  | 26 | OU |  | OU | OU | OU | |  | | OU | OU | OU | OU |
|  | 27 | OU |  | OU | OU | OU | |  | | OU | OU | OU | OU |
