# Supplementary material for: Contrasting impacts of competition on ecological and social trait evolution in songbirds
Source: PLoS Biol. 2018 Jan 31;16(1):e2003563. doi: 10.1371/journal.pbio.2003563 (PMC5809094; doi:10.1371/journal.pbio.2003563)
Supplement: S4 Table — If the model term remained significant after the Bonferroni correction, the sign of the model estimate is presented along with information on the simulation scenarios in which the statistical significance occurred (i.e., BM versus OU null simulations as well as the Szymkiewicz-Simpson coefficient threshold used for sympatry [5%, 20%, 50%, 80%, or all four]). Models for which there is evidence for sympatric convergence, even after controlling for similarity in habitat and A, diet (for models fit to members of the same habitat) or B, habitat (for models fit to species with the same diet) or C, both diet and habitat (for models fit to year-round territorial species), are highlighted in bold. BM, Brownian motion; OU, Ornstein-Uhlenbeck. (DOCX) [file pbio.2003563.s022.docx]

**S4 Table.** Results from pairwise regression analyses. If the model term remained significant after the Bonferroni correction, the sign of the model estimate is presented along with information on the simulation scenarios in which the statistical significance occurred (i.e., BM vs. OU null simulations as well as the Szymkiewicz-Simpson coefficient threshold used for sympatry [5%, 20%, 50%, 80%, or all four]). Models for which there is evidence for sympatric convergence, even after controlling for similarity in habitat and A. diet (for models fit to members of the same habitat) or B. habitat (for models fit to species with the same diet) or C. both diet and habitat (for models fit to year-round territorial species), are highlighted in bold.

|  |  | **female plumage** | | | **male plumage** | | | **song** | | |
| --- | --- | --- | --- | --- | --- | --- | --- | --- | --- | --- |
|  |  | **est.** | **BM** | **OU** | **est.** | **BM** | **OU** | **est.** | **BM** | **OU** |
| ***A. habitat*** | | | | | | | | | | |
| dense | |  |  |  |  |  |  |  |  |  |
|  | sympatry |  |  |  |  |  |  |  |  |  |
|  | diet | - | all | all | - |  | all |  |  |  |
| semi-open | |  |  |  |  |  |  |  |  |  |
|  | sympatry |  |  |  |  |  |  | **-** | **5-20** | **5-50** |
|  | diet |  |  |  |  |  |  | + | all |  |
| open | |  |  |  |  |  |  |  |  |  |
|  | sympatry |  |  |  | + | 50 |  |  |  |  |
|  | diet |  |  |  |  |  |  | - |  | all |
| ***B. diet*** | | | | | | | | | | |
| omnivores | |  |  |  |  |  |  |  |  |  |
|  | sympatry |  |  |  |  |  |  |  |  |  |
|  | habitat | - |  | all | - | all | all |  |  |  |
| frugivores | |  |  |  |  |  |  |  |  |  |
|  | sympatry |  |  |  |  |  |  |  |  |  |
|  | habitat | - |  | all |  |  |  |  |  |  |
| insectivores | |  |  |  |  |  |  |  |  |  |
|  | sympatry |  |  |  |  |  |  | **-** | **5-20** | **5-50** |
|  | habitat |  |  |  |  |  |  |  |  |  |
| granivores | |  |  |  |  |  |  |  |  |  |
|  | sympatry |  |  |  |  |  |  |  |  |  |
|  | habitat |  |  |  |  |  |  |  |  |  |
| ***C. year-round territorial*** | | | | | | | | | | |
|  | sympatry |  |  |  |  |  |  |  |  |  |
|  | diet |  |  |  |  |  |  |  |  |  |
|  | habitat |  |  |  |  |  |  |  |  |  |
