# Supplementary material for: Contrasting impacts of competition on ecological and social trait evolution in songbirds
Source: PLoS Biol. 2018 Jan 31;16(1):e2003563. doi: 10.1371/journal.pbio.2003563 (PMC5809094; doi:10.1371/journal.pbio.2003563)
Supplement: S5 Table — Resource-use traits were set as the reference group so that plumage and song variables test for main effect differences between these traits and resource-use traits. ME, measurement error. (DOCX) [file pbio.2003563.s023.docx]

**S5 Table.** Multiple linear regression analyses of the effect of trait type on statistical support for models incorporating competition, controlling for the effect of measurement error. Resource-use traits were set as the reference group so that plumage and song variables test for main effect differences between these traits and resource-use traits.

|  | **model term** | **estimate** | **std. error** | **t-value** | **p-value** |
| --- | --- | --- | --- | --- | --- |
| ***A. relative support for a model with competition (all data)*** | | | | | |
|  | intercept (resource) | 0.47 | 0.04 | 11.8 | < 0.001 |
|  | median ME | -0.14 | 0.06 | -2.5 | 0.015 |
|  | plumage vs. resource | -0.22 | 0.05 | -4.6 | < 0.001 |
|  | song vs. resource | -0.27 | 0.06 | -4.5 | < 0.001 |
| ***B. relative support for a model with competition (median measurement error < 0.7)*** | | | | | |
|  | intercept (resource) | 0.53 | 0.06 | 8.2 | < 0.001 |
|  | median ME | -0.27 | 0.18 | -1.5 | 0.13 |
|  | plumage vs. resource | -0.24 | 0.05 | -4.4 | < 0.001 |
|  | song vs. resource | -0.24 | 0.08 | -2.9 | 0.005 |
