# Supplementary material for: Contrasting impacts of competition on ecological and social trait evolution in songbirds
Source: PLoS Biol. 2018 Jan 31;16(1):e2003563. doi: 10.1371/journal.pbio.2003563 (PMC5809094; doi:10.1371/journal.pbio.2003563)
Supplement: S6 Table — The effect of this relationship does not depend on the level of statistical support for the DDexp model (i.e., the interaction term is not significant). DDexp, exponential diversity-dependent model; ME, measurement error; MLE, maximum likelihood estimate. (DOCX) [file pbio.2003563.s024.docx]

**S6 Table.** Multiple linear regression analyses of the effect of measurement error on the maximum likelihood estimate of the slope parameter (*r*) of the DD_exp_ model. The effect of this relationship does not depend on the level of statistical support for the DD_exp_ model (i.e., the interaction term is not significant).

|  | **model term** | **estimate** | **std. error** | **t-value** | **p-value** |
| --- | --- | --- | --- | --- | --- |
| ***A. MLE of slope parameter from DD_exp_ model (all data)*** | | | | | |
|  | intercept | 0.04 | 0.006 | 6.2 | < 0.001 |
|  | median ME | 0.02 | 0.008 | 2.9 | 0.004 |
|  | Akaike weight for DD_exp_ | -0.02 | 0.03 | -0.8 | 0.43 |
|  | median ME: Akaike weight for DD_exp_ | 0.10 | 0.08 | 1.2 | 0.22 |
| ***B. MLE of slope parameter from DD_exp_ model (median measurement error < 0.7)*** | | | | | |
|  | intercept | 0.03 | 0.01 | 2.5 | 0.02 |
|  | median ME | 0.07 | 0.03 | 2.7 | 0.008 |
|  | Akaike weight for DD_exp_ | -0.001 | 0.03 | -0.04 | 0.97 |
|  | median ME: Akaike weight for DD_exp_ | 0.03 | 0.10 | 0.3 | 0.79 |
